# Neuroprotective effects and mechanistic insights of sinomenine in ischemic stroke: a systematic review and meta-analysis of preclinical animal studies

**DOI:** 10.3389/fphar.2026.1926226

**Published:** 2026-09-22

**Authors:** Xiaoqun Li, Lulu Wen, Yun Du, Xinzuo Qin, Junyi Zhou, Ningyu Xue, Xinyue Fan, Haiyu Zhao, Miao Qu

**Affiliations:** 1 Third Clinical Medical College of Beijing University of Chinese Medicine, Beijing, China; 2 Department of Neurology, Xuanwu Hospital of Capital Medical University, Beijing, China; 3 Institute of Chinese Materia Medica, China Academy of Chinese Medical Sciences, Beijing, China; 4 Department of Chinese Medicine, Xuanwu Hospital of Capital Medical University, Beijing, China

**Keywords:** animal models, ischemic stroke, meta-analysis, neuroprotection, sinomenine

## Abstract

**Background:**

Ischemic stroke is a leading cause of long-term disability and mortality worldwide. Despite substantial advances in reperfusion therapy, effective neuroprotective strategies remain limited. Sinomenine, a bioactive alkaloid derived from plants of the Menispermaceae family, has shown neuroprotective effects in preclinical models of ischemic stroke. However, the available preclinical evidence has not been systematically quantified, and the relationship between sinomenine dosage and therapeutic efficacy remains unclear.

**Methods:**

Eight electronic databases were searched from inception to 30 March 2026. The risk of bias, reporting quality, and translational readiness were assessed using the SYRCLE risk-of-bias tool, the ARRIVE 2.0 guidelines, and the Stroke Therapy Academic Industry Roundtable criteria, respectively. Data synthesis and quantitative analyses were performed with RevMan 5.3 and Stata 17.0. Subgroup and sensitivity analyses were conducted for outcomes with high heterogeneity. Additionally, dose-response curves were fitted using restricted cubic spline models.

**Results:**

This meta-analysis included 15 studies. In animal models of ischemic stroke, sinomenine significantly reduced cerebral infarct volume, improved neurological function score (NFS), and preserved blood-brain barrier (BBB) integrity. Sinomenine also downregulated neuroinflammation-related biomarkers, enhanced superoxide dismutase (SOD) activity, and modulated apoptosis-related marker levels. RCS analysis suggested nonlinear dose-response relationships between sinomenine dosage and cerebral infarct volume, NFS, and brain water content. Subgroup analyses revealed that animal strain, comorbidity status, experimental blinding, drug dosage, and timing of administration may influence effect sizes. Trim-and-fill analysis showed that the pooled standardized mean difference (SMD) for cerebral infarct volume attenuated from −3.02 (95% CI [-3.96, −2.08]) to −1.97 (95% CI [-2.93, −1.01]) after adjustment, whereas that for NFS attenuated from −1.57 (95% CI [-2.18, −0.97]) to −1.06 (95% CI [-1.72, −0.40]).

**Conclusion:**

In animal models, sinomenine may mitigate ischemic stroke injury through multiple pathways, with the efficacy following nonlinear dose-response relationships, suggesting its potential as an adjunctive neuroprotective intervention warranting further investigation. However, given the methodological limitations of the included studies, these findings should be interpreted cautiously. To facilitate clinical translation, future studies should incorporate more rigorous and standardized experimental designs to strengthen the evidence base and validate its therapeutic potential.

**Systematic Review Registration:**

https://www.crd.york.ac.uk/PROSPERO/view/CRD420261359581, identifier CRD420261359581.

## 1 Introduction

Stroke is the second leading cause of death and the third leading cause of disability worldwide, with nearly 12 million new cases and 7 million deaths annually (Feigin et al., 2025; Hilkens et al., 2024). Characterized by high rates of incidence, disability, and mortality, it imposes a significant burden on global public health systems (Feigin et al., 2023). Stroke is primarily classified into ischemic and hemorrhagic subtypes. Among them, ischemic stroke is the most common clinical type, accounting for 62.4%–67.7% of all stroke cases (Feigin et al., 2024). The core mechanism of ischemic stroke involves the acute interruption of cerebral arterial blood flow, leading to focal cerebral ischemia and hypoxia (DeLong et al., 2022). This pathological process produces two distinct tissue zones within the affected brain: the ischemic core and the ischemic penumbra. The core zone undergoes irreversible neuronal injury due to severe flow reduction (Pensato et al., 2026). However, the penumbra retains partial blood supply, rendering its neurons potentially salvageable (Pensato et al., 2025). Timely restoration of perfusion to this region may preserve some neurological function, representing the key principle of acute ischemic stroke therapy (Nguyen et al., 2024; Hou and Brenner, 2024). Although current standard therapies have improved outcomes in selected patients, important limitations persist. Intravenous thrombolysis is constrained by a narrow therapeutic window and is associated with hemorrhagic risk (Tsivgoulis et al., 2023; Zubair and Sheth, 2023). Endovascular intervention requires strict patient selection, and vessel re-occlusion may occur postoperatively (Oliveira et al., 2023). Furthermore, the clinical translation of most neuroprotective agents remains suboptimal due to poor blood-brain barrier (BBB) permeability (Frank et al., 2022; Salaudeen et al., 2024). Therefore, the inadequacies of existing treatments have driven an urgent search for novel therapeutic strategies.

According to traditional Chinese medicine theory, pathogenic Wind is considered a key pathogenic factor contributing to the development of stroke (Liu and Zhang, 2020; Fu et al., 2013). *Sinomenium acutum* (SA), a traditional ethnic herb, has been historically used to dispel pathogenic Wind and unblock collaterals according to classical Chinese medical texts (Zhen et al., 2026). The “Bencao Tujing” documented that SA was traditionally applied for disorders associated with pathogenic Wind. The “Bencao Huiyan” further described its use in alleviating limb weakness attributed to pathogenic Wind, while the “Bencao Biandu” recorded its application in treating numbness of the extremities. These historical descriptions correspond well with the neurological manifestations commonly observed after ischemic stroke, including motor impairment and sensory disturbances, providing a rationale for investigating the therapeutic potential of SA in post-stroke neurological dysfunction. In recent years, research into bioactive ingredients from natural medicines that protect against ischemic brain injury has attracted considerable attention (Tao et al., 2020; Liu et al., 2024; Liu et al., 2023). Sinomenine (Figure 1) is the major bioactive alkaloid of SA, and modern pharmacological studies have demonstrated its significant anti-inflammatory, immunomodulatory, and analgesic properties in rheumatoid arthritis (Li D. et al., 2025; Wang et al., 2025). With the advancement of neuropharmacological research, the application potential of sinomenine in central nervous system disorders has been increasingly recognized (Zong et al., 2025). Existing preclinical studies have indicated that sinomenine may exert neuroprotective effects in ischemic stroke through multiple mechanisms, including suppression of neuroinflammation, activation of endogenous antioxidant pathways, and regulation of apoptosis (Hong et al., 2022; Hou et al., 2024; Yang Q. et al., 2025). Sinomenine has also been shown to rapidly penetrate the brain and maintain the integrity of the BBB (Gao et al., 2019; Zhang et al., 2021). These multifaceted pharmacological activities suggest that sinomenine may be a promising candidate for adjunctive neuroprotective therapy in ischemic stroke. However, the overall efficacy and underlying mechanisms of sinomenine remain to be clarified due to the lack of systematic synthesis of preclinical evidence. Moreover, the dose-response relationship has yet to be adequately characterized. Therefore, this study aims to conduct a systematic review and meta-analysis of preclinical animal studies investigating sinomenine in the treatment of ischemic stroke, thereby providing a reference for subsequent mechanistic research and experimental design.

**FIGURE 1:**
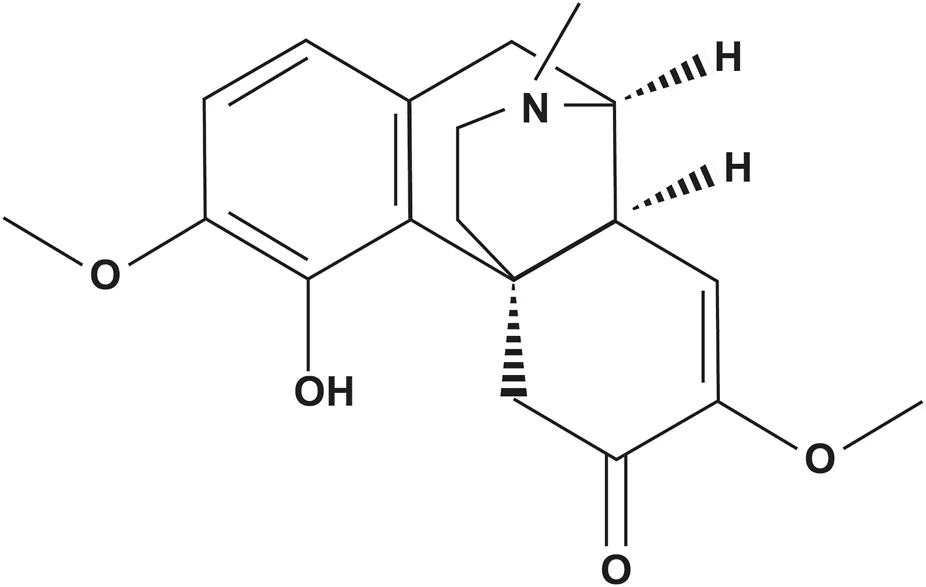
Chemical structure of sinomenine.

## 2 Materials and methods

### 2.1 Study design and registration

This study was designed and conducted in strict adherence to the Preferred Reporting Items for Systematic Reviews and Meta-Analyses (PRISMA) statement (Page et al., 2021). The protocol was prospectively registered with the International Prospective Register of Systematic Reviews (PROSPERO) under registration number CRD420261359581.

### 2.2 Literature search strategy

To comprehensively evaluate the therapeutic effects of sinomenine on ischemic stroke, two reviewers (Xiaoqun Li and Ningyu Xue) independently performed a systematic search of preclinical animal studies across eight electronic databases: Web of Science, PubMed, Embase, the Cochrane Library, China National Knowledge Infrastructure (CNKI), Wanfang Data, the China Science and Technology Journal Database (VIP), and the China Biomedical Literature Service System (Sinomed). The search covered records from database inception to 30 March 2026. A combination of Medical Subject Headings (MeSH) terms and free-text terms was employed. An example search strategy is as follows: (“sinomenine”) AND (“ischemic stroke*” OR “ischaemic stroke*” OR “middle cerebral artery occlusion” OR “MCAO” OR “cerebral ischemia reperfusion”). The search strategy for all databases is detailed in Supplementary Table S1. The language of the literature was not restricted, and studies published in any language were eligible for inclusion.

### 2.3 Inclusion and exclusion criteria

Studies were included if they met the following criteria: (1) Study design: Experimental focal cerebral ischemia models established by the method of transient middle cerebral artery occlusion (tMCAO); (2) Animals: Rodents (rats or mice); (3) Intervention measures: The experimental group received sinomenine monotherapy, whereas the control group was either untreated or treated with vehicle (e.g., normal saline); (4) Outcomes: Primary outcomes included cerebral infarct volume and neurological function score (NFS) (e.g., Zea-Longa score, modified Zea-Longa score, Neurological Severity Score). Secondary outcomes were BBB integrity indicators (brain water content and BBB permeability), neuroinflammatory biomarkers [tumor necrosis factor-α (TNF-α), TNF-α mRNA, interleukin-1β (IL-1β), IL-1β mRNA, myeloperoxidase (MPO), intercellular adhesion molecule-1 (ICAM-1), ionized calcium-binding adapter molecule-1 (Iba1), glial fibrillary acidic protein (GFAP)], oxidative stress markers [superoxide dismutase (SOD)], and apoptosis-related indicators [the number of terminal deoxynucleotidyl transferase (TdT)-mediated dUTP-biotin nick end labeling (TUNEL)-positive cells, B-cell lymphoma 2 (Bcl-2), Bcl-2/Bcl-2-associated X protein (Bax) ratio, caspase-3].

Studies were excluded based on the following criteria: (1) Animal studies employing non-ischemic stroke models (e.g., hemorrhagic stroke, traumatic brain injury) or global cerebral ischemia models; (2) *In vitro* studies; (3) Studies that failed to report any of the aforementioned primary or secondary outcomes; (4) Studies from which effect sizes could not be extracted or calculated; (5) Reviews, conference abstracts, case reports, editorials, and duplicate publications.

### 2.4 Study selection and data extraction

Two reviewers independently performed study selection and data extraction, with cross-checking at each stage. Duplicate records were first removed. Titles and abstracts were then screened to exclude clearly ineligible studies. Potentially eligible records subsequently underwent full-text review to determine final inclusion. Discrepancies were resolved by a third reviewer through arbitration. Data extraction encompassed the following items: first author, publication year, animal characteristics, modeling method, ischemia duration, drug dosage, route of administration, timing of administration, control group treatment, outcome measures, and methodological details. Standard deviation (SD) was calculated from standard error of the mean (SEM) by multiplying the latter by the square root of the sample size (Sedgwick, 2015). For data presented graphically, corresponding authors were first contacted to request raw data. When raw data were unavailable, numerical values were extracted from graphs using WebPlotDigitizer software. Data from 6 studies were extracted using this method. Image digitization was independently performed by 2 reviewers, and the mean of their extracted values was used as the analysis data. The intraclass correlation coefficient (ICC) was used to assess inter-extractor reliability. The overall ICC was 0.997, indicating excellent consistency between the 2 reviewers.

### 2.5 Assessment of study quality and translational readiness

Study quality was assessed across 10 domains using the Systematic Review Centre for Laboratory Animal Experimentation (SYRCLE) risk of bias tool: random sequence generation, baseline characteristics, allocation concealment, random housing, blinding of participants and personnel, random of outcome assessment, blinding of outcome assessment, incomplete outcome data, selective reporting, and other bias (Hooijmans et al., 2014). Each item was rated as low risk, high risk, or unclear risk.

The reporting quality of the included studies was further evaluated using the Essential 10 items of the Animal Research: Reporting of *In Vivo* Experiments 2.0 (ARRIVE 2.0) guidelines (Percie du Sert et al., 2020). The items assessed comprised study design, sample size, inclusion and exclusion criteria, randomization, blinding, outcome measures, statistical methods, experimental animals, experimental procedures, and results.

We evaluated the translational readiness of the included studies according to the preclinical research recommendations proposed by the Stroke Therapy Academic Industry Roundtable (STAIR) (Feinklestein et al., 1999; Fisher et al., 2009). The evaluated items included study design quality, dose-response relationship and therapeutic time window exploration, ischemia model selection, outcome assessment, translational relevance, use of clinically relevant biomarkers, and conflict of interest disclosure.

The above assessments were independently performed by two reviewers. Any disagreements were resolved by consultation with a third reviewer.

### 2.6 Statistical analysis

All outcome measures included in this study were continuous variables. Effect sizes were expressed as standardized mean difference (SMD) with 95% confidence interval (CI). Heterogeneity was quantified using the I^2^ statistic. A fixed-effects model was applied when *I* ^*2*^ < 50% (indicating low heterogeneity), whereas a random-effects model was used when *I* ^*2*^ ≥ 50% (indicating significant heterogeneity). Subgroup analyses were subsequently conducted to explore potential sources of heterogeneity. Sensitivity analysis was conducted using a leave-one-out method to evaluate the robustness of the results. When at least 10 studies were included, funnel plots were constructed and Egger’s test was employed to evaluate publication bias. All statistical analyses were conducted using RevMan 5.3 and Stata 17.0. To investigate the dose-response relationship between sinomenine and relevant efficacy indicators, a restricted cubic spline (RCS) model was fitted. A nonlinearity test was then performed to assess whether the dose-response curve deviated from linearity. Additionally, three-dimensional (3D) scatter plots and radar charts illustrating dose-time-response interactions were generated using Origin 2024. A two-tailed *P*-value <0.05 was considered statistically significant.

## 3 Results

### 3.1 Study selection and characteristics of included studies

The database search initially identified 258 records. After removing 141 duplicates, 117 records underwent title and abstract screening. Of these, 78 records were excluded as clearly irrelevant to the study topic, leaving 39 records for full-text assessment. Following full-text review, 24 studies were excluded for failing to meet the predefined inclusion criteria. The reasons were as follows: inappropriate study design, ineligible animal models, *in vitro* studies, inability to extract quantitative data, and duplicate publication. Ultimately, 15 studies were included in this analysis (Feng et al., 2010; Li et al., 2010; Li et al., 2009; Liang et al., 1999; Wu et al., 2010a; Zhou et al., 2009a; Zhou et al., 2009b; Bi et al., 2021; Li et al., 2026; Qiu et al., 2016a; Qiu et al., 2016b; Wu et al., 2011; Yang et al., 2016; Li et al., 2008; Wu et al., 2010b). The study selection process is summarized in Figure 2.

**FIGURE 2:**
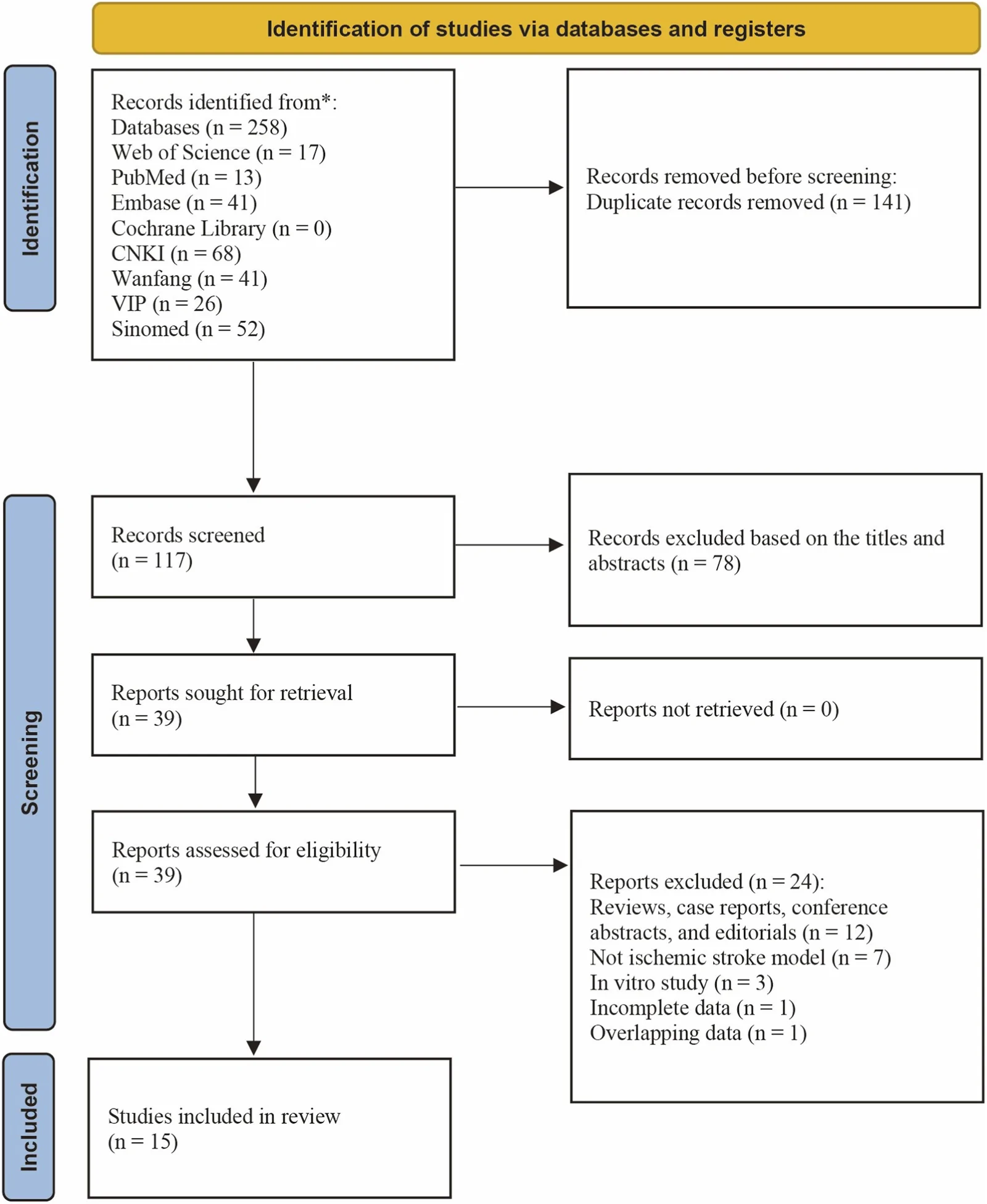
Study selection flowchart.

A total of 15 animal studies investigating sinomenine for ischemic stroke were included. The majority of studies used rats, specifically Wistar rats in 8 studies and Sprague-Dawley (SPD) rats in 4 studies, while the remaining 3 studies used C57BL/6 mice (Figure 3A). All studies reported animal body weight, and 6 reported animal age. Male animals were used in 14 studies, and 1 study did not specify sex (Figure 3B). All studies employed the tMCAO model to replicate ischemic stroke pathology. Among the included studies, 5 established ischemic stroke models in experimental animals with comorbid diabetes, whereas the remainder utilized healthy animals. Sinomenine was administered at doses ranging from 10 to 90 mg/kg for periods of 1–7 days (Figures 3C,D). The intraperitoneal route was used in 14 studies, and tail vein injection in 1 study (Figure 3E). The timing of administration was categorized as pre- or post-MCAO surgery (Figure 3F). For control groups, 12 studies used normal saline, and 3 used untreated controls. Primary and secondary outcome measures are detailed in Table 1. Information on the source and purity of sinomenine is provided in Supplementary Table S2.

**FIGURE 3:**
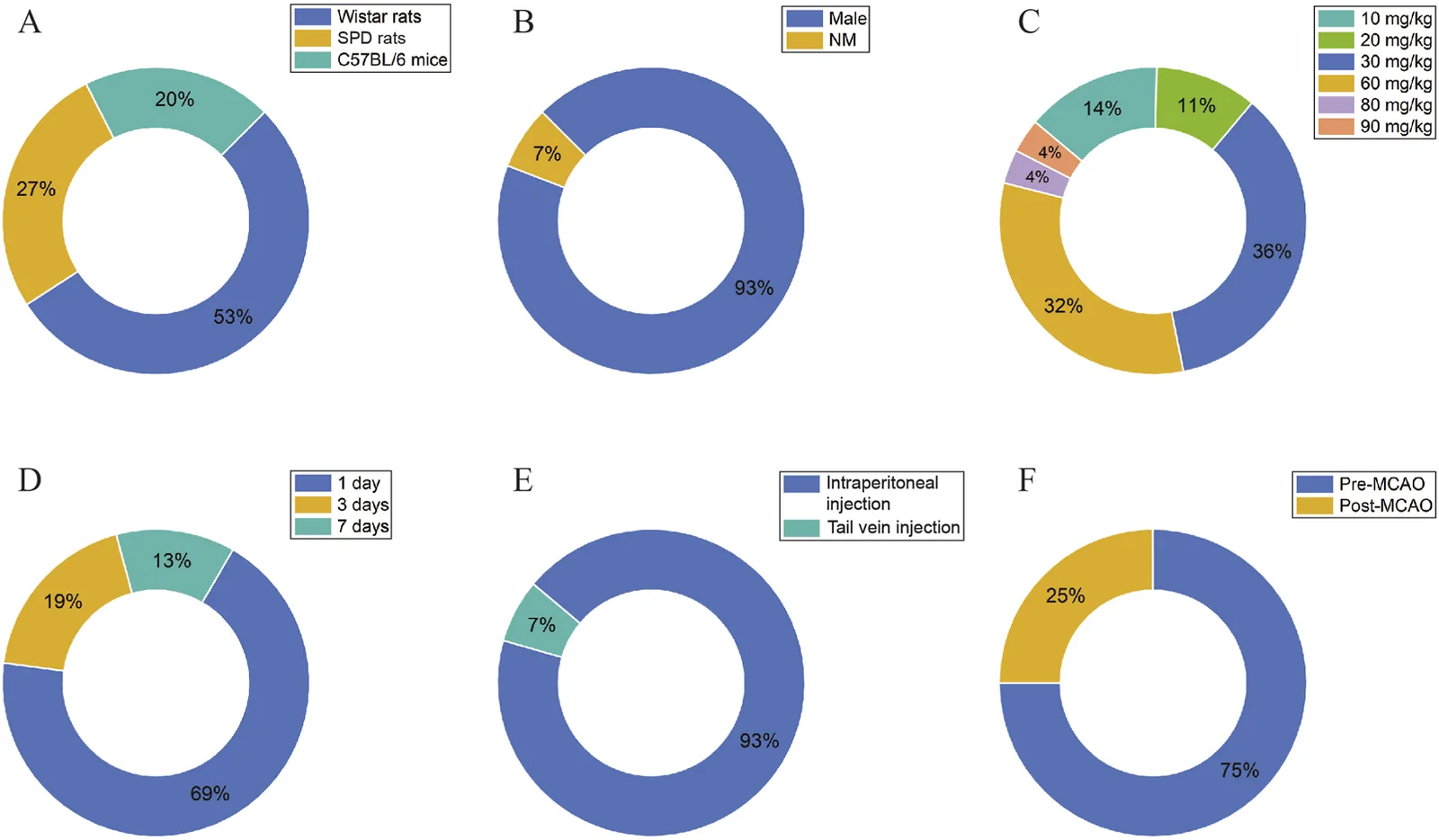
Study characteristics of eligible studies. **(A)** Animal strains. **(B)** Animal sex. **(C)** Drug dosage. **(D)** Duration of intervention. **(E)** Route of administration. **(F)** Timing of administration. SPD rats, Sprague-Dawley rats; MCAO, middle cerebral artery occlusion; NM, not mentioned.

**TABLE 1:** Basic characteristics of the included studies.

Study	Animal characteristics	Model	Experimental group	Control group	Route	Duration	Treatment point	Outcome measures	Major targeting pathophysiology
Feng et al. (2010)	Male Wistar rats (250 ± 30 g)	tMCAO (90 min)	Sinomenine, 30/60 mg/kg	Saline	Intraperitoneal injection	1 day	Pre-MCAO	Cerebral infarct volume, NFS (Zea-Longa score), brain water content	Neuroinflammation
Li et al. (2010)	Male Wistar rats (250 ± 30 g)	tMCAO (90 min)	Sinomenine, 30/60 mg/kg	Saline	Intraperitoneal injection	1 day	Pre-MCAO	Brain water content, MPO, SOD, TUNEL	Neuroinflammation, oxidative stress, apoptosis
Li et al. (2009)	Male Wistar rats (250 ± 30 g)	tMCAO (90 min)	Sinomenine, 30/60 mg/kg	Saline	Intraperitoneal injection	1 day	Pre-MCAO	MPO, ICAM-1	Neuroinflammation
Liang et al. (1999)	Male SPD rats (262 ± 23 g)	tMCAO (120 min)	Sinomenine, 10/30/60 mg/kg	No treatment	Intraperitoneal injection	1 day	Pre-MCAO	Cerebral infarct volume, NFS (modified Zea-Longa score), brain water content	NM
Wu et al. (2010a)	Male Wistar rats (250 ± 30 g) with diabetes	tMCAO (90 min)	Sinomenine, 30/60 mg/kg	Saline	Intraperitoneal injection	1 day	Pre-MCAO	Cerebral infarct volume, Bcl-2, caspase-3	Apoptosis
Zhou et al.(2009a)	Male Wistar rats (250 ± 30 g) with diabetes	tMCAO (90 min)	Sinomenine, 30/60 mg/kg	Saline	Intraperitoneal injection	1 day	Pre-MCAO	Cerebral infarct volume, ICAM-1	Neuroinflammation
Zhou et al.(2009b)	Male Wistar rats (250 ± 30 g) with diabetes	tMCAO (90 min)	Sinomenine, 30/60 mg/kg	Saline	Intraperitoneal injection	1 day	Pre-MCAO	Bcl-2, Bcl-2/Bax	Apoptosis
Bi et al. (2021)	Male C57BL/6 mice (12 weeks, 25 ± 2 g)	tMCAO (60 min)	Sinomenine, 20 mg/kg	No treatment	Intraperitoneal injection	3 days	Post-MCAO	Brain water content, SOD, TNF-α mRNA, IL-1β mRNA	Neuroinflammation, oxidative stress
Li et al. (2026)	Male SPD rats (8–10 weeks, 200–250 g) with diabetes	tMCAO (30 min)	Sinomenine, 80 mg/kg	No treatment	Intraperitoneal injection	7 days	Pre-MCAO	NFS (modified Zea-Longa score)	NM
Qiu et al. (2016a)	Male C57BL/6 mice (8–12 weeks, 20–25 g)	tMCAO (60 min)	Sinomenine, 10/20 mg/kg	Saline	Intraperitoneal injection	3 days	Post-MCAO	Cerebral infarct volume, NFS (Zea-Longa score), brain water content, TNF-α, IL-1β, Iba1, GFAP, caspase-3	Neuroinflammation, apoptosis
Qiu et al. (2016b)	C57BL/6 mice (adult, 20–25 g)	tMCAO (60 min)	Sinomenine, 10/20 mg/kg	Saline	Intraperitoneal injection	3 days	Post-MCAO	Cerebral infarct volume, NFS (Zea-Longa score), brain water content, TNF-α mRNA, IL-1β mRNA, Iba1, GFAP, TUNEL	Neuroinflammation, apoptosis
Wu et al. (2011)	Male SPD rats (3 months, 230–280 g)	tMCAO (120 min)	Sinomenine, 10/30 mg/kg	Saline	Intraperitoneal injection	1 day/7 days	Pre-MCAO/Post-MCAO	Cerebral infarct volume, NFS (Zea-Longa score), Bcl-2/Bax, caspase-3	Apoptosis
Yang et al. (2016)	Male SPD rats (7–9 weeks, 250–290 g)	tMCAO (120 min)	Sinomenine, 90 mg/kg	Saline	Tail vein injection	1 day	Pre-MCAO	Cerebral infarct volume, NFS (neurological severity score), brain water content, BBB permeability, TNF-α, IL-1β, ICAM-1	Neuroinflammation, apoptosis
Li et al. (2008)	Male Wistar rats (250 ± 30 g)	tMCAO (90 min)	Sinomenine, 30/60 mg/kg	Saline	Intraperitoneal injection	1 day	Pre-MCAO	BBB permeability	Neuroinflammation
Wu et al. (2010b)	Male Wistar rats (250 ± 30 g) with diabetes	tMCAO (90 min)	Sinomenine, 30/60 mg/kg	Saline	Intraperitoneal injection	1 day	Pre-MCAO	TNF-α	Neuroinflammation

SPD, rats, Sprague-Dawley rats; tMCAO, transient middle cerebral artery occlusion; NFS, neurological function score; MPO, myeloperoxidase; SOD, superoxide dismutase; TUNEL, terminal deoxynucleotidyl transferase (TdT)-mediated dUTP-biotin nick end labeling; ICAM-1, intercellular adhesion molecule-1; Bcl-2, B-cell lymphoma 2; Bax, Bcl-2-associated X protein; IL-1β, interleukin-1β; TNF-α, tumor necrosis factor-α; Iba1, ionized calcium-binding adapter molecule-1; GFAP, glial fibrillary acidic protein; BBB, blood-brain barrier; NM, not mentioned.

### 3.2 Quality and translational readiness assessment results of the included studies

A structured risk of bias assessment was conducted for all 15 included studies (Figures 4A,B). Regarding randomization, 13 studies clearly reported random allocation of animals, while the remaining 2 did not detail the randomization method. All studies reported balanced baseline characteristics; however, none described allocation concealment or randomization of housing. For blinding, only 1 study blinded investigators and personnel, and 4 studies blinded outcome assessors. Seven studies reported randomization of outcome assessment. All other risk of bias domains, including incomplete outcome data, selective reporting, and other potential sources of bias, were rated as low risk.

**FIGURE 4:**
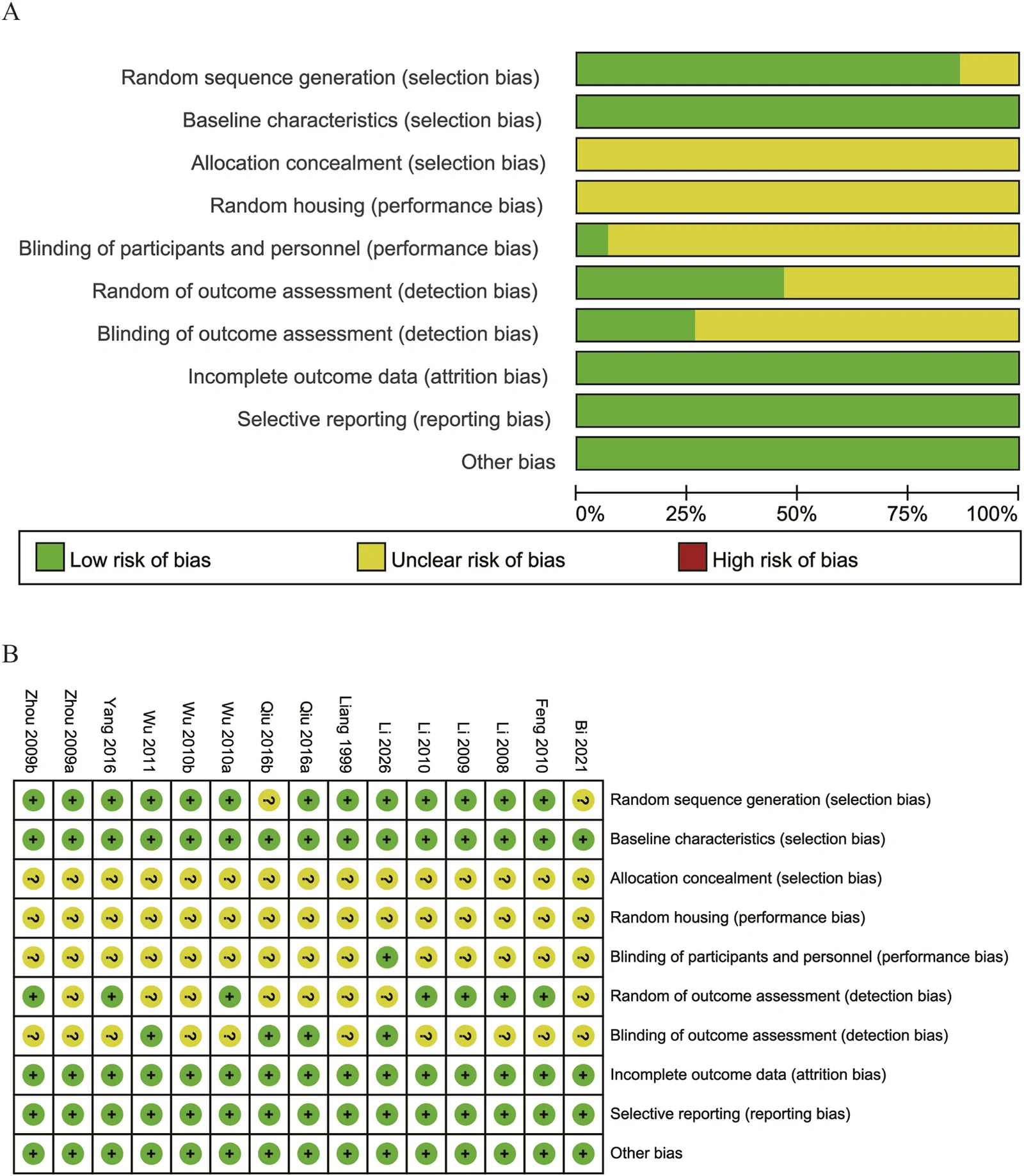
Risk of bias plot of the included studies. **(A)** Risk of bias graph. **(B)** Risk of bias summary.

The completeness of reporting in the included studies was assessed using the ARRIVE 2.0 Essential 10 items (Supplementary Table S3). All 15 studies reported study design, the number of animals used, definitions of outcome measures, characteristics of experimental animals, experimental procedures, and descriptive statistics of the results. Reporting was also relatively complete for animal provenance (14 studies), pre-specified inclusion and exclusion criteria (14 studies), and randomization methods (13 studies). However, several important methodological details were inadequately reported. Only 8 studies reported whether any animals were excluded from the analysis; 4 reported blinding; 2 described acclimatization periods; and only 1 reported an *a priori* sample size calculation. None of the included studies described strategies used to minimize potential confounders, specified the primary outcome measure, or reported an effect size with a CI.

The translational readiness of the included studies was assessed based on the STAIR recommendations, with the results summarized in Supplementary Table S4. Regarding study design quality, 13 studies reported randomization procedures, 4 implemented blinding, 14 predefined inclusion and exclusion criteria, and 8 reported animal exclusions. Only 1 study reported an *a priori* sample size calculation, while 5 reported physiological monitoring parameters. Twelve studies investigated dose-response relationships, whereas only 1 explicitly evaluated the therapeutic time window. All included studies used the tMCAO model, and none evaluated both acute and long-term histological and functional outcomes. Regarding translational relevance, none of the studies performed multi-species validation, included aged animals, or conducted reproducibility validation. Fourteen studies used exclusively male animals, 5 incorporated comorbidity models, and 14 employed clinically relevant biomarkers. Conflict of interest statements were provided in 5 studies.

### 3.3 Cerebral infarct volume

Eight animal studies reported cerebral infarct volume data, yielding 17 comparisons. Heterogeneity was significant across studies (*I* ^*2*^ = 76%, *P* < 0.00001). Meta-analysis using a random-effects model demonstrated that sinomenine intervention significantly reduced cerebral infarct volume in animal models of ischemic stroke compared with controls (SMD = −3.02, 95% CI [-3.96, −2.08], *P* < 0.00001; Figure 5A).

**FIGURE 5:**
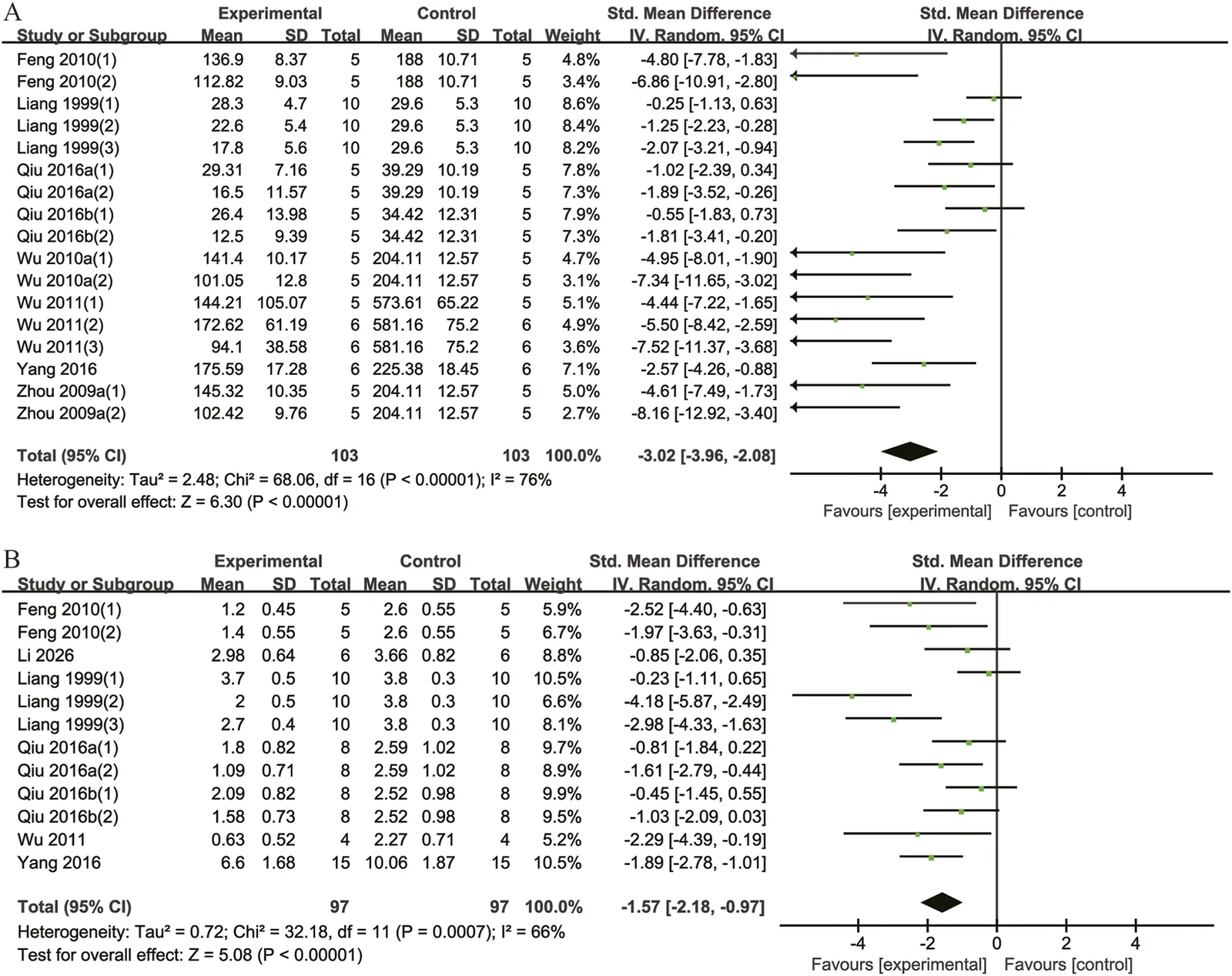
**(A)** Forest plot of cerebral infarct volume. **(B)** Forest plot of NFS.

### 3.4 NFS

Seven animal studies assessed NFS, generating 12 comparisons. Significant heterogeneity was observed among these studies (*I* ^*2*^ = 66%, *P* = 0.0007). Pooled analysis using a random-effects model showed that sinomenine significantly reduced NFS relative to controls (SMD = −1.57, 95% CI [-2.18, −0.97], *P* < 0.00001; Figure 5B).

### 3.5 BBB functional markers

Brain water content and BBB permeability are key indicators of BBB integrity. Significant heterogeneity was observed for both outcomes (brain water content: *I* ^*2*^ = 67%, *P* = 0.0003; BBB permeability: *I* ^*2*^ = 86%, *P* = 0.0006). Pooled analysis from the random-effects model indicated that sinomenine protected against post-ischemic BBB disruption by reducing brain water content (SMD = −2.47, 95% CI [-3.28, −1.66], *P* < 0.00001; Figure 6A) and decreasing BBB permeability (SMD = −7.50, 95% CI [-14.87, −0.14], *P* = 0.05; Figure 6B).

**FIGURE 6:**
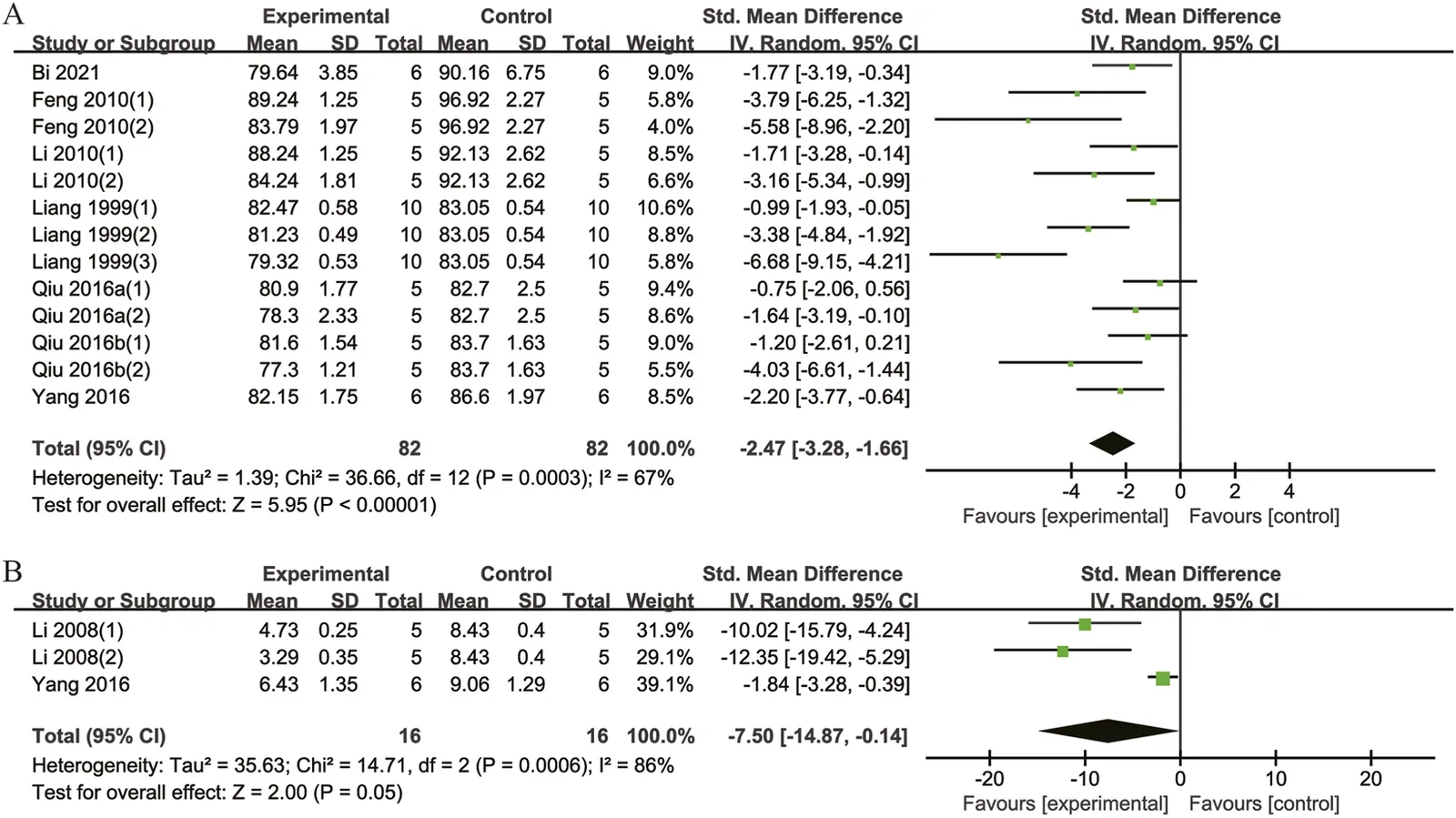
**(A)** Forest plot of brain water content. **(B)** Forest plot of BBB permeability.

### 3.6 Neuroinflammation-related indicators

This meta-analysis investigated the effects of sinomenine intervention on neuroinflammation-related biomarkers, examining inflammatory mediators (TNF-α, TNF-α mRNA, IL-1β, IL-1β mRNA), markers of inflammatory cell infiltration (MPO, ICAM-1), and glial activation markers (Iba1, GFAP). Heterogeneity testing revealed no significant heterogeneity between studies for any of the inflammatory mediators: TNF-α (*I* ^*2*^ = 19%, *P* = 0.29), TNF-α mRNA (*I* ^*2*^ = 0%, *P* = 0.48), IL-1β (*I* ^*2*^ = 49%, *P* = 0.16), and IL-1β mRNA (*I* ^*2*^ = 0%, *P* = 0.54). Fixed-effects meta-analysis demonstrated that sinomenine intervention significantly reduced TNF-α protein levels (SMD = −1.75, 95% CI [-2.65, −0.84], *P* = 0.0001; Figure 7A), TNF-α mRNA levels (SMD = −1.65, 95% CI [-2.77, −0.52], *P* = 0.004; Figure 7B), IL-1β protein levels (SMD = −1.49, 95% CI [-2.86, −0.13], *P* = 0.03; Figure 7C), and IL-1β mRNA levels (SMD = −1.34, 95% CI [-2.39, −0.29], *P* = 0.01; Figure 7D) in experimental animals.

**FIGURE 7:**
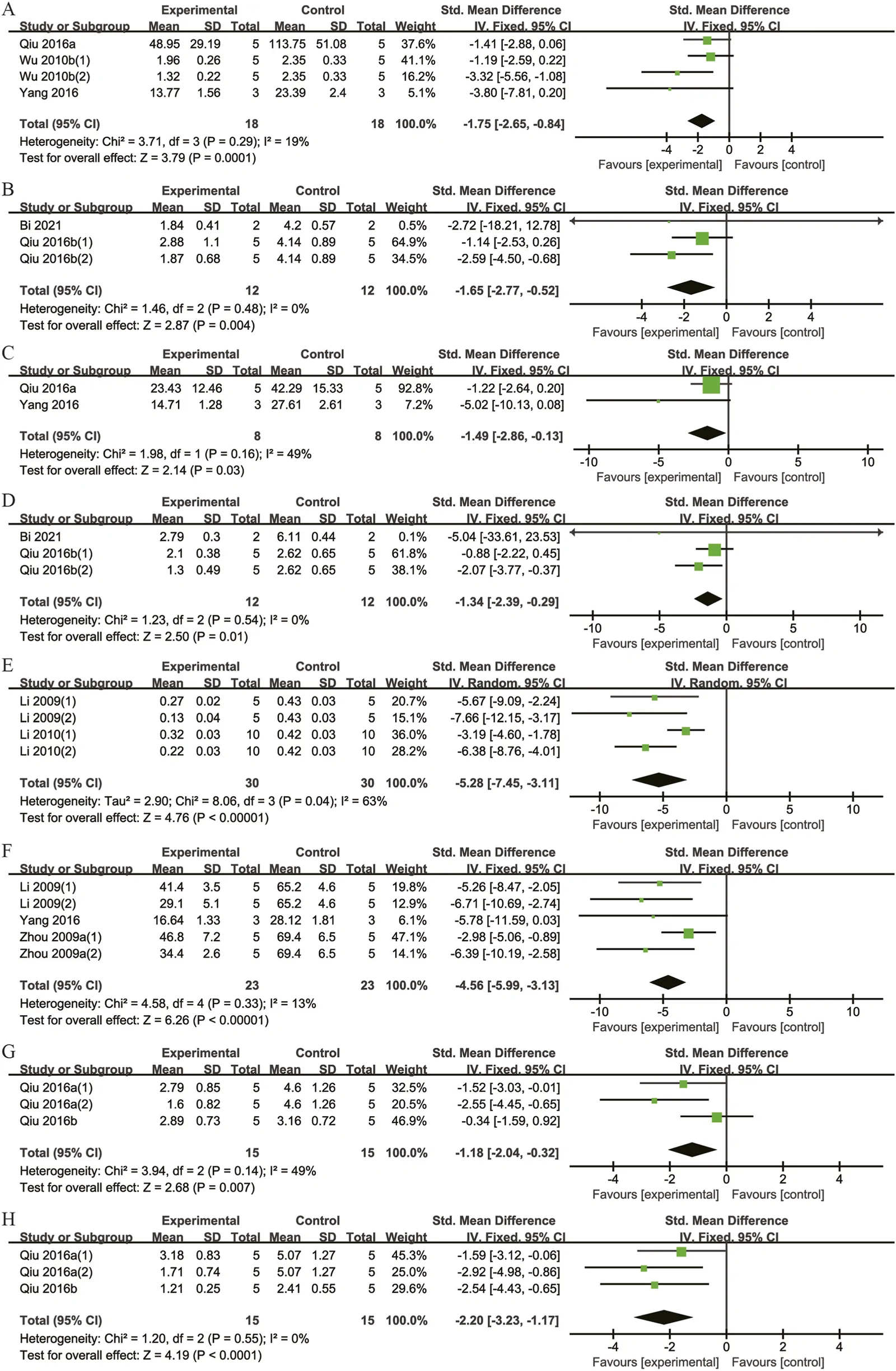
**(A)** Forest plot of TNF-α level. **(B)** Forest plot of TNF-α mRNA level. **(C)** Forest plot of IL-1β level. **(D)** Forest plot of IL-1β mRNA level. **(E)** Forest plot of MPO level. **(F)** Forest plot of ICAM-1 level. **(G)** Forest plot of Iba1 level. **(H)** Forest plot of GFAP level.

Significant heterogeneity was observed across studies for MPO levels (*I* ^*2*^ = 63%, *P* = 0.04), whereas no significant heterogeneity was detected for ICAM-1 (*I* ^*2*^ = 13%, *P* = 0.33). Accordingly, a random-effects model and a fixed-effects model were applied, respectively. Analysis revealed that sinomenine significantly decreased both MPO levels (SMD = −5.28, 95% CI [-7.45, −3.11], *P* < 0.00001; Figure 7E) and ICAM-1 levels (SMD = −4.56, 95% CI [-5.99, −3.13], *P* < 0.00001; Figure 7F). No significant heterogeneity was identified across studies for Iba1 (*I* ^*2*^ = 49%, *P* = 0.14) or GFAP (*I* ^*2*^ = 0%, *P* = 0.55). Fixed-effects meta-analysis revealed that sinomenine significantly lowered Iba1 levels (SMD = −1.18, 95% CI [-2.04, −0.32], *P* = 0.007; Figure 7G) and GFAP levels (SMD = −2.20, 95% CI [-3.23, −1.17], *P* < 0.0001; Figure 7H) in experimental animals.

### 3.7 Oxidative stress-related indicators

Two studies (involving 3 comparisons) evaluated changes in SOD levels in animal models of ischemic stroke. Heterogeneity analysis revealed no significant between-study heterogeneity (*I* ^*2*^ = 46%, *P* = 0.16). Meta-analysis using a fixed-effects model demonstrated that sinomenine intervention significantly increased SOD levels in experimental animals (SMD = 3.02, 95% CI [2.02, 4.01], *P* < 0.00001; Figure 8A).

**FIGURE 8:**
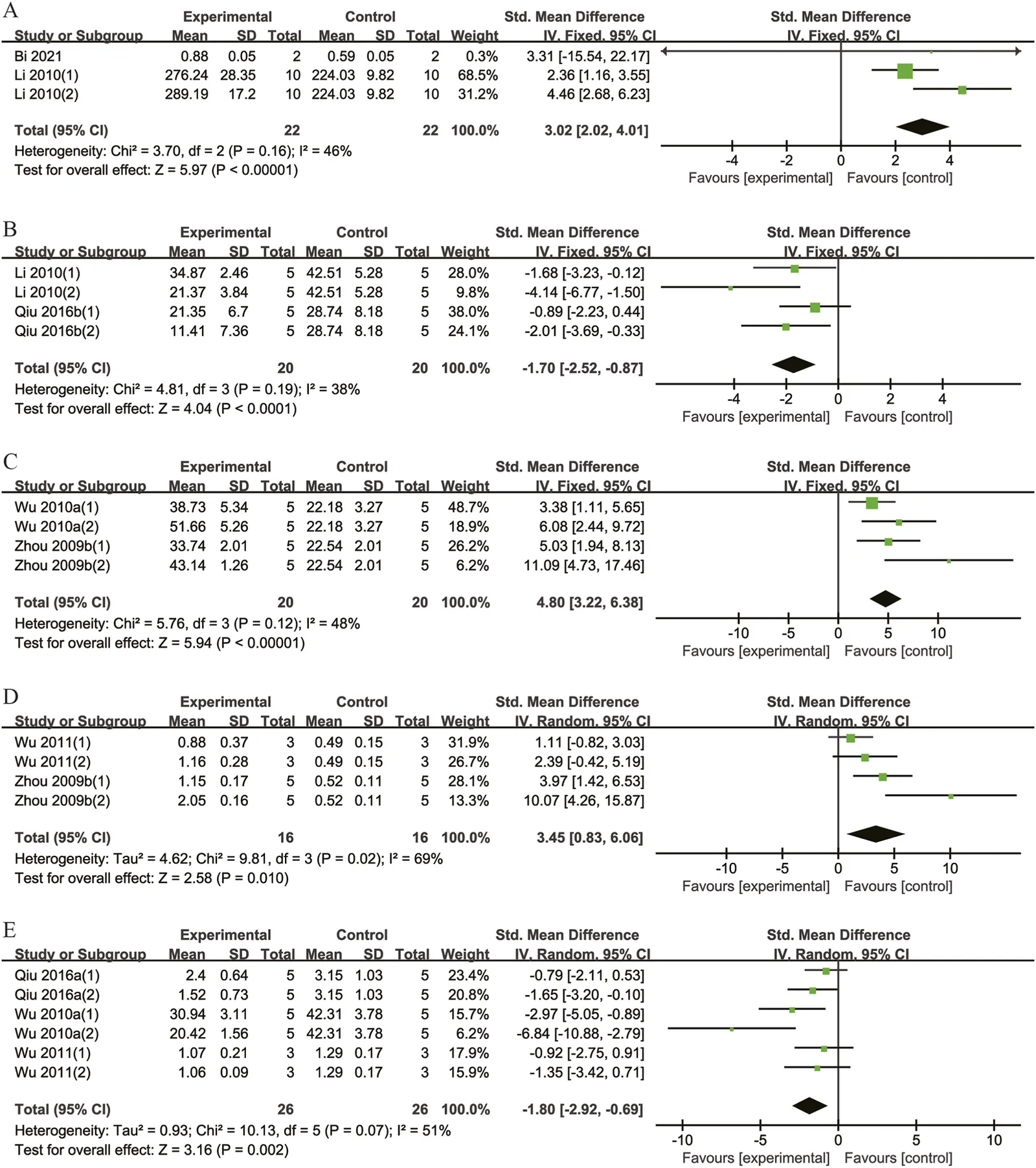
**(A)** Forest plot of SOD. **(B)** Forest plot of TUNEL-positive cells. **(C)** Forest plot of Bcl-2 level. **(D)** Forest plot of Bcl-2/Bax ratio. **(E)** Forest plot of caspase-3 level.

### 3.8 Apoptosis-related indicators

This meta-analysis evaluated the effects of sinomenine on apoptosis, focusing on TUNEL-positive cell counts, Bcl-2 levels, the Bcl-2/Bax ratio, and caspase-3 levels. No significant heterogeneity was detected among studies reporting TUNEL-positive cell counts (*I* ^*2*^ = 38%, *P* = 0.19) or Bcl-2 levels (*I* ^*2*^ = 48%, *P* = 0.12); therefore, a fixed-effects model was applied. Significant between-study heterogeneity was detected for the Bcl-2/Bax ratio (*I* ^*2*^ = 69%, *P* = 0.02) and caspase-3 levels (*I* ^*2*^ = 51%, *P* = 0.07), necessitating analysis with a random-effects model. Pooled results showed that sinomenine significantly reduced the number of TUNEL-positive cells (SMD = −1.70, 95% CI [-2.52, −0.87], *P* < 0.0001; Figure 8B), increased Bcl-2 levels (SMD = 4.80, 95% CI [3.22, 6.38], *P* < 0.00001; Figure 8C) and the Bcl-2/Bax ratio (SMD = 3.45, 95% CI [0.83, 6.06], *P* = 0.010; Figure 8D), and downregulated caspase-3 levels (SMD = −1.80, 95% CI [-2.92, −0.69], *P* = 0.002; Figure 8E).

### 3.9 Dose-response analysis

To further elucidate the relationship between sinomenine dosage and neuroprotective effects, this study employed an RCS model for dose-response analysis. RCS modeling revealed that the dose-response relationships between sinomenine and cerebral infarct volume (*χ* ^*2*^ = 10.48, *P* = 0.001), NFS (*χ* ^*2*^ = 17.34, *P* < 0.001), and brain water content (*χ* ^*2*^ = 14.71, *P* = 0.0001) all exhibited significant nonlinearity (Figures 9A–C). Specifically, with increasing sinomenine dose, both cerebral infarct volume and brain water content exhibited nonlinear downward trends that gradually approached saturation; whereas NFS displayed a pronounced bell-shaped curve, with the maximal therapeutic effect observed at approximately 44.40 mg/kg. These findings suggested that, within a certain dose range, the effects of sinomenine on these 3 outcomes might be dose-dependent. Furthermore, descriptive 3D and radar plots demonstrated that sinomenine administered at 20–90 mg/kg improved relevant outcome measures in animal models of ischemic stroke over a 1- to 3-day treatment period (Figures 10A–F).

**FIGURE 9:**
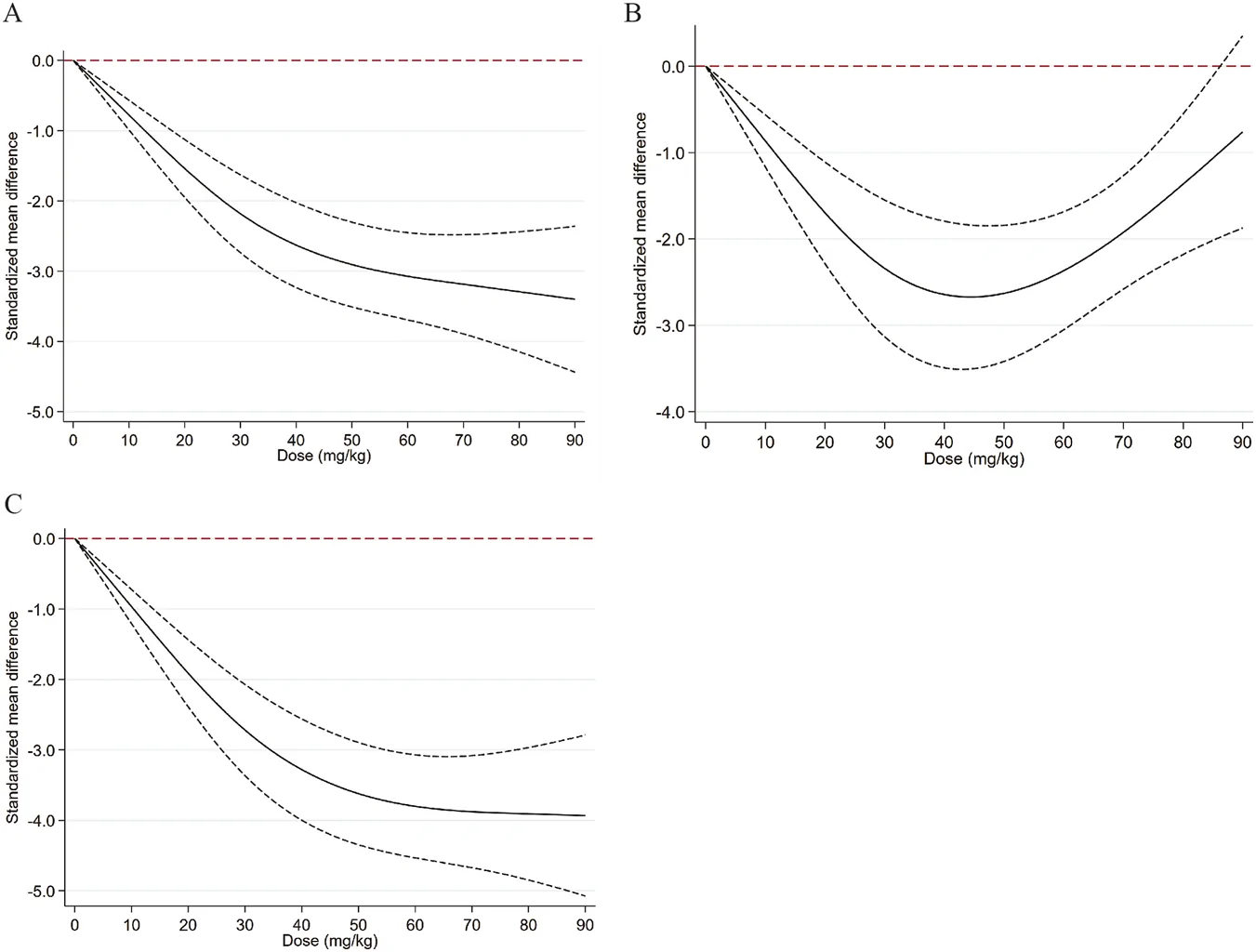
**(A)** Dose-response curve of sinomenine on cerebral infarct volume. **(B)** Dose-response curve of sinomenine on NFS. **(C)** Dose-response curve of sinomenine on brain water content.

**FIGURE 10:**
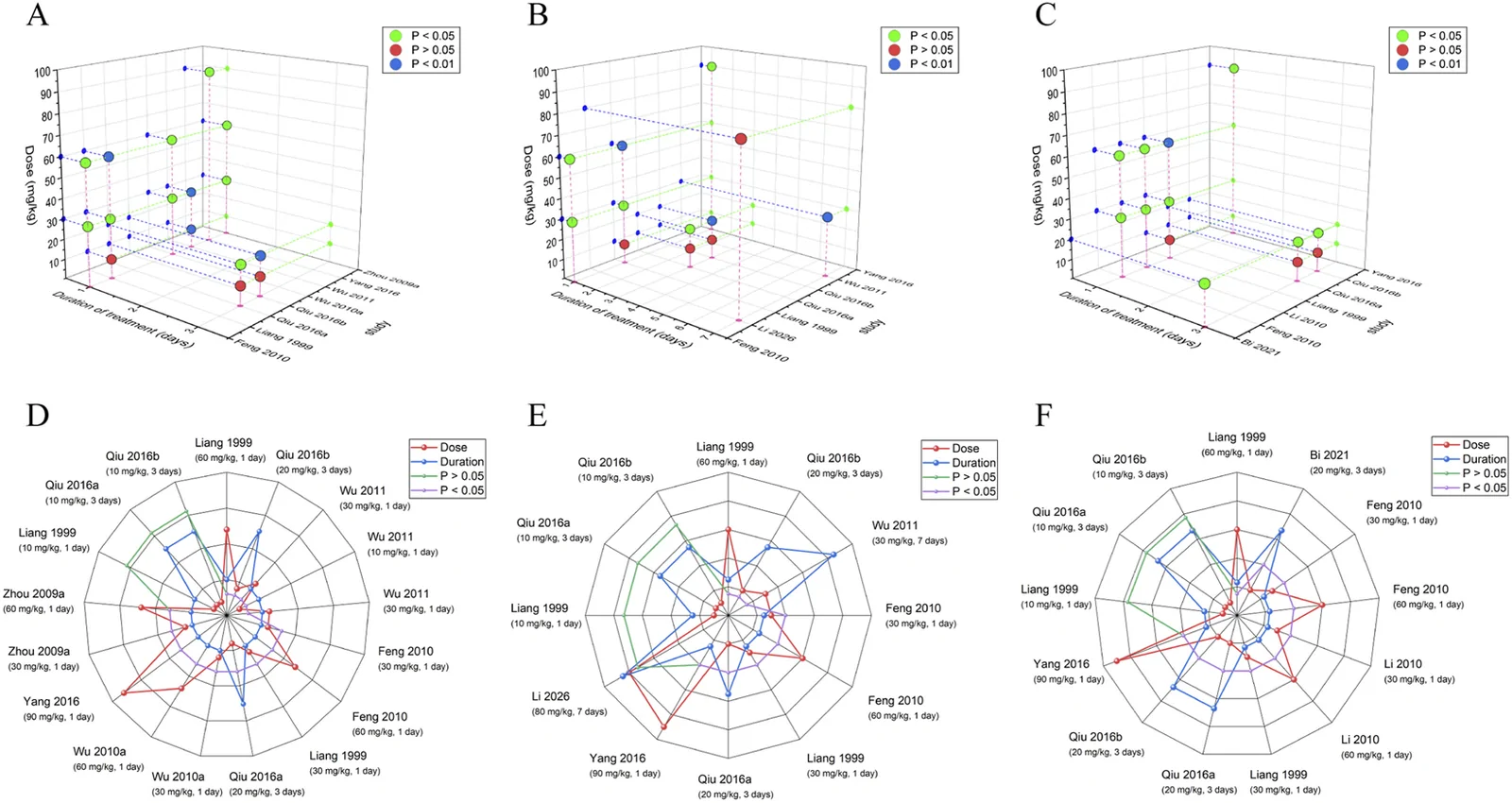
**(A)** 3D scatter plot of cerebral infarct volume. **(B)** 3D scatter plot of NFS. **(C)** 3D scatter plot of brain water content. **(D)** Radar chart of cerebral infarct volume. **(E)** Radar chart of NFS. **(F)** Radar chart of brain water content.

### 3.10 Subgroup analysis

We conducted subgroup analyses of cerebral infarct volume, NFS, and brain water content (Supplementary Table S5-S7). The stratification variables included animal strain, comorbidity status, experimental blinding, sinomenine dosage, and timing of administration.

Subgroup analysis of cerebral infarct volume revealed that animal strain (*I* ^*2*^ = 93.4%, *P* < 0.00001), comorbidity status (*I* ^*2*^ = 90.7%, *P* = 0.001), dosage (*I* ^*2*^ = 58.0%, *P* = 0.05), and timing of administration (*I* ^*2*^ = 87.2%, *P* = 0.005) were likely the primary sources of heterogeneity between studies (Supplementary Figures S1-S4). No heterogeneity was detected among studies regarding experimental blinding (*I* ^*2*^ = 0%, *P* = 0.44) (Supplementary Figure S5). Sinomenine significantly reduced cerebral infarct volume across all animal strain subgroups, including C57BL/6 mice (SMD = −1.20, 95% CI [-1.92, −0.48]), SPD rats (SMD = −2.69, 95% CI [-4.05, −1.34]), and Wistar rats (SMD = −5.61, 95% CI [-7.02, −4.19]). Compared with healthy animal models (SMD = −2.37, 95% CI [-3.27, −1.47]), sinomenine demonstrated a more pronounced reduction in cerebral infarct volume in animal models with comorbid diabetes (SMD = −5.65, 95% CI [-7.41, −3.90]). The effect of sinomenine on reducing cerebral infarct volume may be influenced by blinding: blinded studies reported a smaller pooled effect size (SMD = −2.65, 95% CI [-4.07, −1.23]) than non-blinded studies (SMD = −3.42, 95% CI [-4.77, −2.07]). Regarding dosage, significant neuroprotective effects were observed at doses of 20 mg/kg and above. Moreover, the pooled effect size increased progressively as the dosage rose from 10 to 60 mg/kg. The maximal effect size was observed at 60 mg/kg (SMD = −5.70, 95% CI [-9.38, −2.03]), whereas the effect was slightly diminished at 90 mg/kg (SMD = −2.57, 95% CI [-4.26, −0.88]). Both pre-MCAO administration (SMD = −3.94, 95% CI [-5.30, −2.58]) and post-MCAO administration (SMD = −1.56, 95% CI [-2.53, −0.59]) of sinomenine significantly reduced cerebral infarct volume, with pre-MCAO yielding a larger effect size.

For NFS, subgroup analyses indicated that animal strain (*I* ^*2*^ = 61.8%, *P* = 0.07), experimental blinding (*I* ^*2*^ = 72.9%, *P* = 0.05), dosage (*I* ^*2*^ = 80.1%, *P* = 0.0001), and timing of administration (*I* ^*2*^ = 65.6%, *P* = 0.09) may be the principal sources of heterogeneity (Supplementary Figures S6-S9). Sinomenine significantly improved neurological function in ischemic stroke models across all animal strains: C57BL/6 mice (SMD = −0.93, 95% CI [-1.46, −0.40]), SPD rats (SMD = −1.96, 95% CI [-3.08, −0.83]), and Wistar rats (SMD = −2.21, 95% CI [-3.46, −0.96]). Regarding NFS, sinomenine produced significant improvements in both blinded (SMD = −0.98, 95% CI [-1.46, −0.51]) and non-blinded (SMD = −2.19, 95% CI [-3.33, −1.05]) studies, with the latter exhibiting a more pronounced effect. With respect to dosage, all subgroups except 10 mg/kg (SMD = −0.47, 95% CI [-1.02, 0.09]) and 80 mg/kg (SMD = −0.85, 95% CI [-2.06, 0.35]) demonstrated protective effects, with the most pronounced benefits observed at 30 mg/kg (SMD = −3.11, 95% CI [-4.32, −1.89]) and 60 mg/kg (SMD = −2.58, 95% CI [-3.63, −1.53]). Both subgroups defined by administration timing showed neuroprotective potential, with pre-MCAO administration yielding superior therapeutic efficacy (SMD = −1.98, 95% CI [-2.96, −0.99]).

Subgroup analysis of brain water content suggested that between-study heterogeneity was primarily attributed to differences in animal strain (*I* ^*2*^ = 56.8%, *P* = 0.10), experimental blinding (*I* ^*2*^ = 68.5%, *P* = 0.08), dosage (*I* ^*2*^ = 75.7%, *P* = 0.002), and timing of administration (*I* ^*2*^ = 77.1%, *P* = 0.04) (Supplementary Figures S10-S13). Sinomenine significantly reduced brain water content in C57BL/6 mice (SMD = −1.54, 95% CI [-2.34, −0.75]), SPD rats (SMD = −3.09, 95% CI [-5.08, −1.09]), and Wistar rats (SMD = −3.14, 95% CI [-4.63, −1.65]). Regarding brain water content, non-blinded studies demonstrated a larger pooled effect size (SMD = −2.91, 95% CI [-3.99, −1.83]) than blinded studies (SMD = −1.54, 95% CI [-2.59, −0.49]). All dosage subgroups demonstrated statistically significant effect sizes. The effect of sinomenine on reducing brain water content strengthened with increasing dosage, peaking at 60 mg/kg (SMD = −5.03, 95% CI [-7.33, −2.73]). Additionally, reductions in brain water content were observed with both pre-MCAO administration (SMD = −3.13, 95% CI [-4.38, −1.88]) and post-MCAO administration (SMD = −1.54, 95% CI [-2.34, −0.75]), with a larger effect size for the former.

### 3.11 Sensitivity analysis and publication bias

Sensitivity analysis was performed using the leave-one-out method for cerebral infarct volume, NFS, and brain water content. The pooled effect sizes for these outcomes remained stable after exclusion of any single study, indicating robustness of the results (Supplementary Figure S14). Furthermore, to address the potential non-independence among the eight included studies (Feng et al., 2010; Li et al., 2010; Li et al., 2009; Wu et al., 2010a; Zhou et al., 2009a; Zhou et al., 2009b; Li et al., 2008; Wu et al., 2010b), which exhibited high consistency in experimental animals, model parameters, and intervention protocols, we additionally performed a sensitivity analysis (Supplementary Figures S15-S16). After excluding these studies, the pooled effect size for cerebral infarct volume decreased to −1.99 (95% CI [-2.83, −1.14]), and that for the NFS decreased to −1.49 (95% CI [-2.16, −0.82]). Although the effect sizes for both outcomes were reduced relative to the primary analysis, the direction of effect remained unchanged. This suggests that the effect sizes reported in the primary analysis may have been overestimated due to the potential non-independence among these studies.

Publication bias was assessed for cerebral infarct volume, NFS, and brain water content (Supplementary Table S8). The funnel plot for cerebral infarct volume revealed an asymmetric distribution of effect sizes among included studies, and Egger’s test indicated significant publication bias (*P* < 0.001) (Figures 11A,B). Following trim-and-fill correction, the pooled effect size changed from −3.02 (95% CI [-3.96, −2.08]) to −1.97 (95% CI [-2.93, −1.01]) (Supplementary Figure S17A). Likewise, the funnel plot for NFS showed asymmetry, with Egger’s test yielding *P* = 0.023 (Figures 11C,D). Following trim-and-fill correction, the pooled effect size was adjusted from −1.57 (95% CI [-2.18, −0.97]) to −1.06 (95% CI [-1.72, −0.40]) (Supplementary Figure S17B). The funnel plot for brain water content was also asymmetric, with Egger’s test showing *P* < 0.001 (Figures 11E,F). Trim-and-fill correction shifted the pooled effect size from −2.47 (95% CI [-3.28, −1.66]) to −1.93 (95% CI [-2.83, −1.04]) (Supplementary Figure S17C). In summary, all three outcome measures exhibited evidence of publication bias, with statistically significant Egger’s test results. Notably, trim-and-fill analysis revealed that after imputing hypothetical studies, the pooled effect size was attenuated but remained directionally consistent, suggesting that the original effect of sinomenine may be overestimated. Therefore, while the available evidence indicated a favorable trend for sinomenine in improving the aforementioned outcomes, a cautious interpretation of its neuroprotective efficacy remained warranted.

**FIGURE 11:**
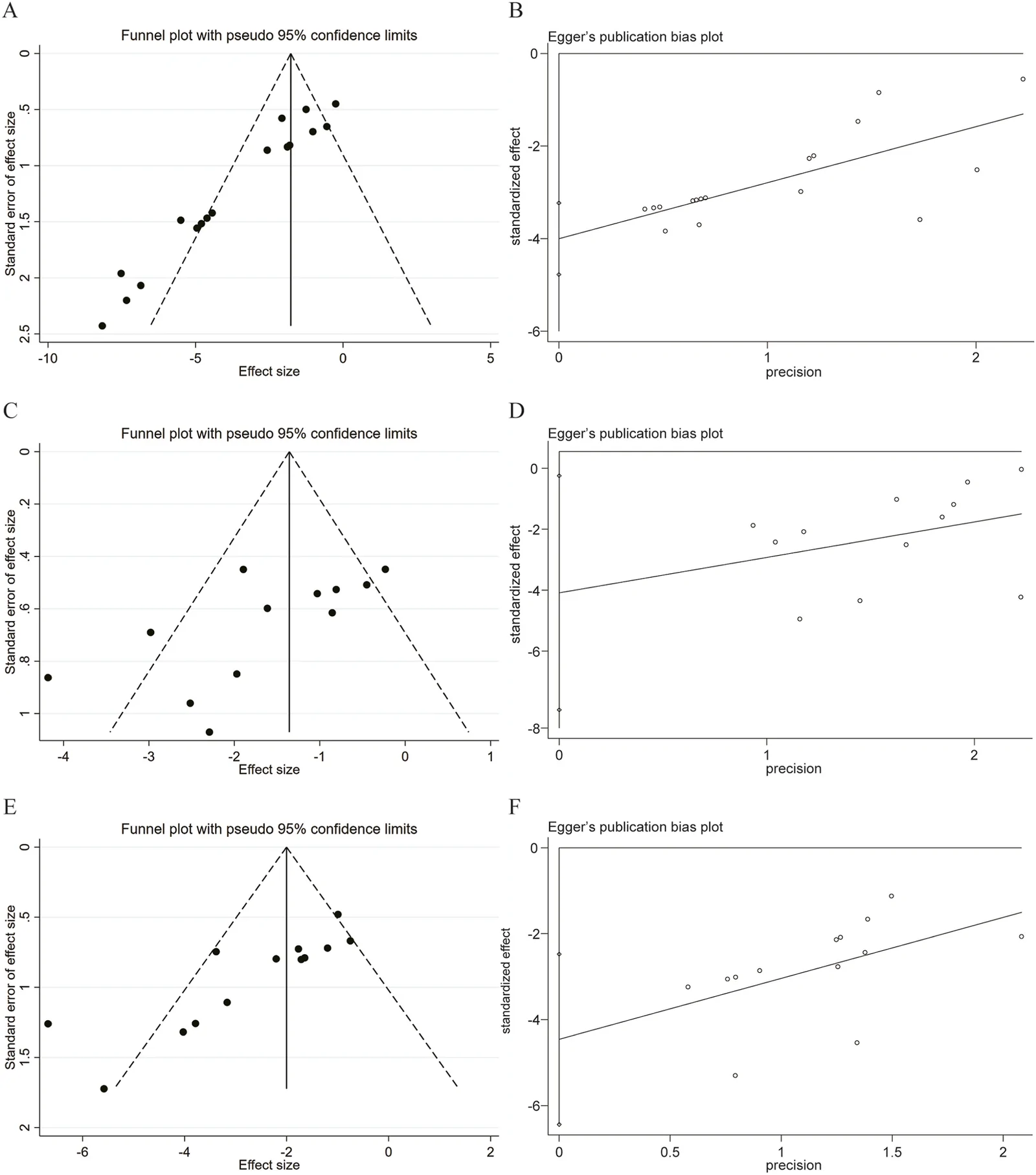
**(A)** Funnel plot of cerebral infarct volume. **(B)** Egger’s plot of cerebral infarct volume. **(C)** Funnel plot of NFS. **(D)** Egger’s plot of NFS. **(E)** Funnel plot of brain water content. **(F)** Egger’s plot of brain water content.

## 4 Discussion

### 4.1 Summary of evidence

Following its initial isolation by the Japanese scholar Ishiwari in the 1920s, the pharmacological activity of sinomenine in inflammatory and painful conditions has garnered increasing interest (Yamasaki, 1976). Pharmacokinetic studies demonstrate that following peripheral administration, sinomenine is widely distributed to multiple tissues, including the kidneys, liver, heart, brain, and testicles (Wang et al., 2023; Li X. et al., 2025). Notably, sinomenine can achieve therapeutically relevant concentrations in brain tissue, indicating its capacity to cross the BBB (Wu et al., 2011; Zhao et al., 2012). This property enables sinomenine to act on the central nervous system, thereby expanding its research value and therapeutic potential for neurological disorders. Therefore, by integrating published preclinical animal data, this study presents the first comprehensive and systematic evaluation of sinomenine’s pharmacological effects and potential mechanisms in ischemic stroke models.

The results of the meta-analysis indicated that sinomenine significantly reduced cerebral infarct volume and NFS. The pooled effect sizes for both outcomes were relatively large, suggesting robust biological relevance of its neuroprotective effects. Similarly, BBB function parameters showed significant pooled effect sizes. Sinomenine attenuated cerebral edema and suppressed the abnormal increase in BBB permeability, thereby preventing secondary neurotoxicity triggered by the entry of peripheral inflammatory cells and harmful substances into the brain parenchyma. Neuroinflammation suppression appears to be a key mechanism underlying sinomenine-mediated neuroprotection. The responses of microglia and astrocytes following ischemic stroke are bidirectional: at different pathological stages, these two glial cell types can mediate either tissue damage or exert protective effects (Yang S. et al., 2025; Candelario-Jalil et al., 2022; Williamson et al., 2021). During the acute phase of cerebral ischemia, persistently activated glial cells undergo pro-inflammatory phenotypic remodeling and release inflammatory factors and neurotoxic molecules, thereby further exacerbating secondary brain injury (Alsbrook et al., 2023). Therefore, sinomenine-induced downregulation of Iba1 and GFAP expression within this window may primarily suppress ischemia-induced pathological glial overactivation and neuroinflammation, rather than interfere with their intrinsic protective or late-stage repair functions. Moreover, the reduction in the pooled effect size of pro-inflammatory cytokines (TNF-α, IL-1β) provided further support for this interpretation. In addition, sinomenine significantly decreased the expression of neutrophil infiltration-associated markers MPO and ICAM-1. The effects of sinomenine on oxidative stress levels should be interpreted cautiously. The meta-analysis revealed increased SOD activity based on only 3 comparisons, which somewhat diminishes the evidence level of the pooled result. The meta-analysis of apoptosis-related indicators further confirmed that sinomenine modulated ischemia-induced neuronal apoptosis through multiple effects. These included upregulation of the anti-apoptotic protein Bcl-2, increase in the Bcl-2/Bax ratio, inhibition of executioner caspase-3 activation, and reduction in TUNEL-positive cell counts. The comprehensive quality assessment using the SYRCLE tool and the ARRIVE 2.0 guidelines revealed that the included studies were limited in both methodological rigor and reporting transparency. Evaluation based on the STAIR criteria indicated that preclinical studies on sinomenine still face several limitations to achieving clinical translation, particularly regarding multi-species validation, long-term functional evaluation, animal model representativeness, and experimental reproducibility verification.

In summary, the preclinical evidence synthesized in this meta-analysis suggests that sinomenine exerts neuroprotective effects in animal models of ischemic stroke. Neuroprotective strategies have increasingly been investigated as potential adjuncts to reperfusion therapy for ischemic stroke (Fisher and Savitz, 2022; Zhao et al., 2024). In the context of ischemia-reperfusion injury, the pooled evidence supports further evaluation of sinomenine as a potential adjunctive neuroprotective therapy for ischemic stroke.

### 4.2 Interpretation of dose-response findings

The RCS model revealed a potential nonlinear association between sinomenine dosage and its therapeutic efficacy. The RCS curves indicated that, at doses below approximately 60 mg/kg, the reducing effects of sinomenine on cerebral infarct volume and brain water content were enhanced with increasing dose, exhibiting a typical dose-dependent pattern; however, once the dose exceeded this threshold, the curves plateaued, suggesting a possible dose plateau effect. Sinomenine-induced improvement in neurological function initially increased but subsequently decreased as the dose rose, reaching the maximal effect at approximately 44.40 mg/kg. This suggested that an optimal dose window might exist regarding neurological function protection. Descriptive analysis revealed that sinomenine was potentially effective within a dose range of 20–90 mg/kg, and that short-term administration for 1–3 days tended to exert neuroprotective effects. A previous study reported that following intraperitoneal administration of 30 mg/kg sinomenine to rats, the linear detection ranges in plasma and brain tissue were 0.1–100 μg/mL and 0.01–5.00 μg/g, respectively (Long et al., 2010). *In vitro* experiments using PC12 cells demonstrated that sinomenine at concentrations of 0.060–0.600 μg/mL attenuated H_2_O_2_-induced cellular injury (Long et al., 2010). This *in vitro* effective concentration was consistent with measured drug levels in brain tissue, indicating that a 30 mg/kg dose could achieve a biologically active concentration in the brain. It is noteworthy that this dose falls within the effective range shown in the present study. Intraperitoneal administration of 10 mg/kg sinomenine yielded a brain concentration of 0.11 ± 0.04 μg/g in healthy SPD rats, whereas a higher dose (30 mg/kg) significantly reduced cerebral infarct volume and ameliorated neurological deficits in rats with focal cerebral ischemia (Wu et al., 2011). Furthermore, sinomenine was detected in brain tissue following administration at doses of 25 mg/kg or 90 mg/kg (Huang et al., 2022; Liu et al., 2005).

### 4.3 Mechanistic insights into neuroprotection

The principal pathological damage in ischemic stroke encompasses neuronal necrosis in the ischemic core and a secondary injury cascade in the penumbra (Dou et al., 2023). Neuroinflammation, oxidative stress, and neuronal apoptosis are the crucial mechanisms mediating secondary brain injury (Lin et al., 2023; Yang M. et al., 2025). As described in Section 4.1, the quantitative meta-analysis in this study demonstrated that sinomenine exerts multi-target protective effects against the aforementioned pathological processes (Figure 12). Building upon these findings, we further synthesized existing literature to comprehensively explore the potential downstream molecular mechanisms that may underlie these effects. It should be noted that the specific signaling pathways discussed below, namely, nuclear factor erythroid 2-related factor 2 (Nrf2), nuclear factor kappa B (NF-κB), NOD-like receptor family pyrin domain-containing 3 (NLRP3), acid-sensing ion channel 1a (ASIC1a), phosphatidylinositol 3-kinase/protein kinase B/mammalian target of rapamycin (PI3K/Akt/mTOR), and mTOR/UNC-51-like kinase 1 (ULK1), are primarily derived from individual experimental studies, and the supporting evidence remains at a narrative rather than a quantitative level.

**FIGURE 12:**
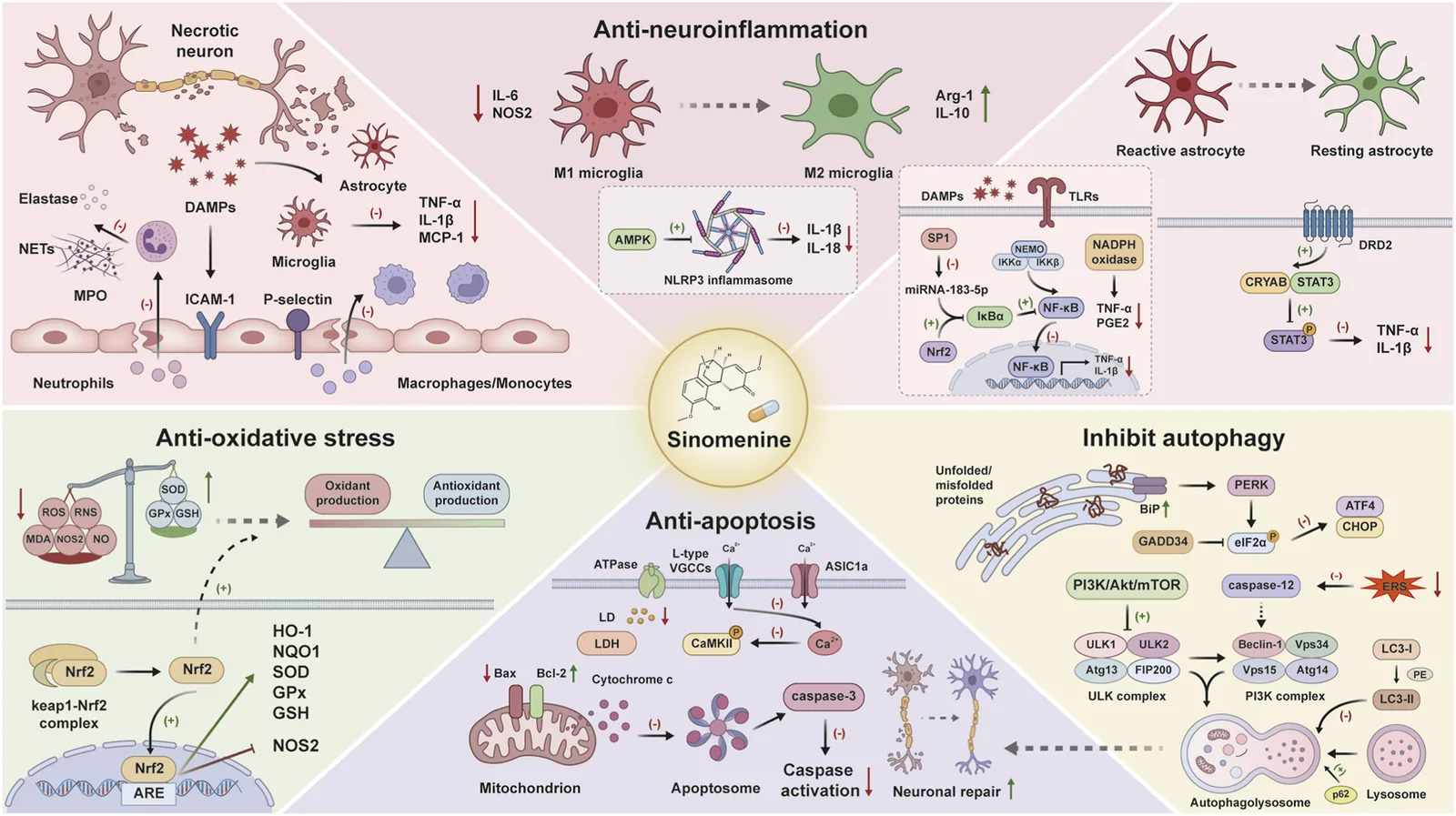
Neuroprotective mechanisms of sinomenine in ischemic stroke. Note: The findings illustrated in the figure—including the inhibition of glial overactivation, reduction in inflammatory infiltration markers (MPO, ICAM-1), decrease in pro-inflammatory cytokines (TNF-α, IL-1β), enhancement of SOD activity, restoration of the Bcl-2/Bax balance, and downregulation of caspase-3—represent the anti-neuroinflammatory, oxidative stress-modulating, and anti-apoptotic effects quantitatively derived from preclinical data. DAMPs, damage-associated molecular patterns; TNF-α, tumor necrosis factor-α; IL-1β, interleukin-1β; MCP-1, monocyte chemotactic protein-1; MPO, myeloperoxidase; ICAM-1, intercellular adhesion molecule-1; NETs, neutrophil extracellular traps; IL-6, interleukin-6; NOS2, inducible nitric oxide synthase; Arg-1, arginase-1; IL-10, interleukin-10; AMPK, AMP-activated protein kinase; NLRP3, NOD-like receptor family pyrin domain-containing 3; TLRs, Toll-like receptors; NF-κB, nuclear factor kappa B; Nrf2, nuclear factor erythroid 2-related factor 2; IκBα, inhibitor of kappa B alpha; IL-18, interleukin-18; NEMO, NF-κB essential modulator; IKKα, IκB kinase alpha; IKKβ, IκB kinase beta; PGE2, prostaglandin E2; SP1, specificity protein 1; DRD2, dopamine D2 receptor; CRYAB, αB-crystallin; STAT3, signal transducer and activator of transcription 3; ROS, reactive oxygen species; RNS, reactive nitrogen species; MDA, malondialdehyde; LDH, lactate dehydrogenase; NO, nitric oxide; GPx, glutathione peroxidase; GSH, glutathione; SOD, superoxide dismutase; ARE, antioxidant response element; NQO1, NAD(P)H: quinone oxidoreductase 1; ASIC1a, acid-sensing ion channel 1a; VGCCs, voltage-gated calcium channels; CaMKII, calcium/calmodulin-dependent protein kinase II; LD, lactic acid; LC3, microtubule-associated protein 1 light chain 3; mTOR, mammalian target of rapamycin; ULK1, UNC-51-like kinase 1; CHOP, C/EBP homologous protein; GADD34, growth arrest and DNA damage-inducible protein 34; BiP, immunoglobulin heavy chain binding protein; PERK, protein kinase R (PKR)-like endoplasmic reticulum kinase; ATF4, activating transcription factor 4; eIF2α, eukaryotic initiation factor 2 alpha; ERS, endoplasmic reticulum stress; PI3K, phosphatidylinositol 3-kinase; Akt, protein kinase B.

#### 4.3.1 Anti-neuroinflammatory effects

Following ischemic stroke, damage-associated molecular patterns (DAMPs) released by damaged neurons activate the innate immune system, rapidly triggering microglial M1 polarization and reactive astrogliosis, and promoting the massive release of inflammatory mediators such as TNF-α and IL-1β (Sakai and Shichita, 2023; Liang et al., 2023). The inflammatory microenvironment induces cerebral microvascular endothelial cells to upregulate the expression of ICAM-1, mediating adhesion and transendothelial migration of peripheral neutrophils (Sienel et al., 2021; Rahmig et al., 2024). Infiltrating neutrophils further exacerbate brain injury by releasing MPO, elastase, and neutrophil extracellular traps (NETs) (Krishnamoorthy et al., 2026). On the basis of the anti-inflammatory effects supported by quantitative results, multiple original studies have explored the molecular mechanisms of sinomenine. One study found that in microglia, sinomenine can inhibit the expression of M1-associated factors such as interleukin-6 (IL-6) and inducible nitric oxide synthase (NOS2) and upregulate M2-associated markers such as arginase-1 (Arg-1) and interleukin-10 (IL-10), and this effect may be related to activation of the Nrf2 signaling pathway, inhibition of inhibitor of kappa B alpha (IκBα) phosphorylation and degradation, and suppression of NF-κB p65 nuclear translocation (Bi et al., 2021). Sinomenine also reduced the production of pro-inflammatory mediators such as TNF-α and prostaglandin E2 (PGE2) by inhibiting the activity of NADPH oxidase in microglia (Qian et al., 2007). Additionally, a study reported that sinomenine inhibited NLRP3 inflammasome activation by modulating AMP-activated protein kinase (AMPK) signaling and reduced the levels of the associated inflammatory cytokines IL-1β and interleukin-18 (IL-18) (Qiu et al., 2016a). However, it should be noted that the role of AMPK in ischemic stroke is context-dependent; its activation may exert either protective or deleterious effects depending on the specific model, cell type, and ischemic stage (Li and McCullough, 2010; Paskeh et al., 2022). Another study indicated that sinomenine can block NF-κB activation by regulating the specificity protein 1 (SP1)/miRNA-183-5p/IκBα axis (Qin et al., 2018). With regard to astrocytes, an individual study has shown that sinomenine activates dopamine D2 receptor (DRD2) to upregulate αB-crystallin (CRYAB), thereby inhibiting signal transducer and activator of transcription 3 (STAT3) phosphorylation and downstream inflammatory gene expression (Qiu et al., 2016b). In terms of neutrophil regulation, sinomenine can reduce MPO activity and inhibit the expression of ICAM-1 and P-selectin, thus decreasing neutrophil accumulation in brain tissue (Li et al., 2009; Zhou et al., 2009a). One *in vivo* study demonstrated that sinomenine downregulates monocyte chemotactic protein-1 (MCP-1) and TNF-α levels to reduce the migration of peripheral monocytes/macrophages into the ischemic brain region (Wu et al., 2010b).

#### 4.3.2 Anti-oxidative stress effects

During ischemia-reperfusion, excessive generation of reactive oxygen species (ROS) and reactive nitrogen species (RNS) overwhelms endogenous antioxidant defenses (Anwar et al., 2025; Lochhead et al., 2024). This leads to lipid peroxidation, protein oxidation and DNA damage, culminating in cell death (Pawluk et al., 2024). Multiple original studies have suggested that sinomenine can effectively intervene in this pathological process. One study in a rat model of cerebral ischemia-reperfusion injury found that sinomenine reversed the elevation of malondialdehyde (MDA) levels and the decline in SOD activity (Li et al., 2010). Another study reported that sinomenine significantly decreased serum lactate dehydrogenase (LDH) and MDA levels while reducing nitric oxide (NO) release in model rats (Yin et al., 2019). This indicated its capacity to ameliorate both cell membrane damage and nitrosative stress (Yin et al., 2019). Furthermore, experiments based on the Nrf2 pathway revealed that sinomenine enhanced glutathione peroxidase (GPx) and SOD activities, thereby restoring cerebral antioxidant capacity (Bi et al., 2021). At the same time, it inhibited NOS2 expression to reduce formation of RNS such as peroxynitrite (Bi et al., 2021). An *in vitro* study demonstrated that sinomenine pretreatment decreased MDA levels and restored both glutathione (GSH) content and SOD activity in PC12 cells following H_2_O_2_ exposure (Fan et al., 2017). Interestingly, this protective effect was not attributable to direct free radical scavenging by sinomenine. Instead, it was mediated by the induction of moderate ROS generation, which subsequently activated the Nrf2 signaling pathway-driven endogenous antioxidant response (Fan et al., 2017). In conjunction with the quantitative analyses from the present study, these findings demonstrate that sinomenine may be associated with alleviating brain tissue damage related to oxidative stress in ischemic stroke models.

#### 4.3.3 Anti-apoptotic effects

During ischemic stroke, neuronal apoptosis occurs in the ischemic penumbra due to hypoxia and impaired energy metabolism (Uzdensky, 2019). Ischemia-induced stress signals such as excitotoxicity and oxidative stress disrupt the balance of Bcl-2 family proteins, leading to mitochondrial release of cytochrome c, which forms the apoptosome with apoptotic protease activating factor-1 (Apaf-1) and activates the caspase cascade (Zhang et al., 2022; Duan et al., 2025). Sinomenine corrected the Bcl-2/Bax imbalance, inhibited the mitochondrial apoptotic pathway, and in mouse MCAO models reduced caspase-3 levels and the number of TUNEL-positive neurons while increasing neuronal survival (Zhou et al., 2009b; Qiu et al., 2016a; Qiu et al., 2016b). Beyond the apoptotic parameters quantitatively validated in this meta-analysis, individual studies have also suggested that sinomenine may exert anti-apoptotic effects through modulation of calcium homeostasis and autophagy. One study found that sinomenine inhibited activities related to ASIC1a and L-type voltage-gated calcium channels (VGCCs), attenuated ischemia-induced calcium influx and calcium overload, and decreased calcium/calmodulin-dependent protein kinase II (CaMKII) phosphorylation levels (Wu et al., 2011). Another study observed that sinomenine downregulated ASIC1a mRNA expression in the ischemic brain tissue of rats, reduced lactic acid (LD) content and LDH activity, and restored ATPase activity (Yang et al., 2016). It is noteworthy that autophagy plays a complex role in neuronal death following ischemia (Shi et al., 2023). Previous studies have shown that excessive autophagy can promote neuronal apoptosis (Mao et al., 2022; Wu et al., 2025; Stanzione et al., 2024). One study reported that sinomenine decreased Beclin-1 expression, reduced the microtubule-associated protein 1 light chain 3 (LC3)-II/LC3-I ratio, and elevated p62 levels, and proposed that the mTOR/ULK1 signaling pathway might be involved in its regulation of aberrant autophagy after cerebral ischemia (Yin et al., 2019). Under hypoxic conditions *in vitro*, sinomenine reduced the expression of C/EBP homologous protein (CHOP) and caspase-12 while increasing growth arrest and DNA damage-inducible protein 34 (GADD34) and immunoglobulin heavy chain binding protein (BiP) levels, indicating its ability to suppress autophagy induced by endoplasmic reticulum stress (ERS) (Bai et al., 2018). This study further revealed that sinomenine activated the PI3K/Akt/mTOR signaling pathway, thereby enhancing its anti-apoptotic effects in neurons (Bai et al., 2018).

### 4.4 Interpretation of subgroup analysis findings

Subgroup analysis across the three rodent strains revealed that sinomenine exerted beneficial effects in all groups. This was confirmed by the reduction in cerebral infarct volume, improvement in neurological function, and alleviation of cerebral edema. Rats exhibit superior integrity of the circle of Willis and more abundant collateral circulation than mice, and their cerebrovascular structure more closely resembles that of humans (Narayan et al., 2021; Fluri et al., 2015). This suggests that rats may offer greater anatomical advantages for modeling human stroke. Wistar and SPD rats are two commonly used rodent strains. However, these strains may differ in susceptibility to cerebral ischemia: Wistar rats demonstrated larger infarct volumes in tMCAO models and more severe cerebral edema in permanent MCAO models than did SPD rats (Walberer et al., 2006). Notably, this study found that the effect sizes for the relevant outcomes followed a graded pattern across strains, with Wistar rats demonstrating the strongest response, SPD rats an intermediate response, and C57BL/6 mice relatively modest effects. These findings suggest that Wistar rats may represent the most suitable strain for validating the efficacy of sinomenine in future studies, as they offer the greatest statistical power for detecting treatment effects.

Diabetes, a common comorbidity of stroke, may serve as a potential effect modifier of sinomenine’s neuroprotective efficacy. Hyperglycemia can exacerbate ischemic brain injury and increase cerebral infarct volume, and may concurrently heighten cerebral tissue susceptibility to ischemia-reperfusion injury through mechanisms including enhanced oxidative stress and neuroinflammation, aggravated vascular dysfunction, and BBB disruption (Kotova et al., 2023; Kilic and Halici, 2025; Hashmat et al., 2025). Consequently, the more severe pathological damage in the context of diabetes may provide greater scope for sinomenine’s neuroprotective intervention, thereby partially accounting for the larger effect size observed in diabetic animals. Nevertheless, this finding warrants further validation, as the diabetic subgroup comprised a limited number of comparisons, and potential confounding factors such as inter-study differences in experimental conditions cannot be entirely ruled out.

Blinding status may have influenced the assessment of sinomenine’s efficacy. Across the three outcome measures (cerebral infarct volume, NFS, and brain water content), the pooled effect sizes in blinded studies were consistently smaller than those in non-blinded studies, suggesting that the absence of blinding may lead to an overestimation of therapeutic efficacy. The lack of blinding in preclinical studies can readily introduce observer bias and expectation bias, thereby exaggerating between-group differences (Bello et al., 2014; Karp et al., 2022). Notably, all outcomes included in the blinding subgroup analysis achieved statistical significance, and the direction of effect was consistent with the overall results. This enhances the reliability of the primary conclusions, indicating that the neuroprotective effect of sinomenine is not solely driven by non-blinded studies of lower methodological quality.

The neuroprotective effects of sinomenine in ischemic stroke may be influenced by dose, although the sensitivity of different outcome measures to dose varied. At the low dose of 10 mg/kg, sinomenine alleviated cerebral edema but produced only minimal improvements in cerebral infarct volume and neurological deficits. This suggests that 10 mg/kg may fail to achieve therapeutically effective concentrations in brain tissue, making it inadequate to block post-ischemic cascades and thus limiting efficacy. At 20 mg/kg, sinomenine produced significant improvements across all three outcomes with low heterogeneity. As the dosage increased to 30 mg/kg, the efficacy for each measure further increased. Within the dose range observed in the subgroup analysis of this study, sinomenine reached its peak effects on cerebral infarct volume and cerebral edema at 60 mg/kg. In the 80 mg/kg group, only one study reported negative outcomes regarding neurological function. However, this result warrants cautious interpretation given the small sample size for this comparison. At 90 mg/kg, effect sizes for all outcomes decreased modestly yet remained statistically significant, suggesting a potential ceiling effect. Therefore, the optimal dose range for sinomenine to exert stable efficacy in animal models of ischemic stroke may be 20–90 mg/kg. It is worth noting that this range aligns with the results of dose-response analysis in this study, further supporting the robustness of our conclusions.

Both pre-MCAO and post-MCAO administration strategies demonstrated therapeutic potential in rodent models of ischemic stroke. Overall, pre-MCAO administration yielded superior efficacy compared with post-MCAO delivery. However, it should be noted that the post-MCAO administration subgroup included notably fewer studies, which may affect the precision of the pooled effect estimate.

### 4.5 Strengths and limitations

Previous reviews have primarily provided narrative summaries of sinomenine’s pharmacological effects and mechanisms within the broader context of cardio-cerebrovascular or central nervous system diseases (Hong et al., 2022; Zhang et al., 2021). The present study focused on animal models of focal cerebral ischemia to quantitatively evaluate its neuroprotective effects via meta-analysis, explore dose-response relationships and potential factors influencing therapeutic efficacy, and assess evidence quality, risk of bias, and the underlying mechanisms. Compared with previous qualitative reviews, this study provides more systematic quantitative evidence for the overall preclinical efficacy of sinomenine in treating ischemic stroke.

However, this meta-analysis has several limitations that necessitate cautious interpretation of the findings. Firstly, the included studies exhibited deficiencies in methodological quality. Most studies provided inadequate reporting of randomization details, allocation concealment, and blinding implementation, increasing the risk of selection, performance, and detection bias. Furthermore, although WebPlotDigitizer is widely used for data extraction in meta-analysis of animal experiments, its accuracy depends on image resolution and reviewer judgment, which may introduce measurement error.

Secondly, the large pooled effect sizes reported for some outcome measures (e.g., cerebral infarct volume: SMD = −3.02; NFS: SMD = −1.57; BBB permeability: SMD = −7.50) should be interpreted cautiously and should not be regarded as direct estimates of the true biological effects of sinomenine. These potentially inflated effects may reflect the combined impact of small sample sizes in preclinical studies, inter-study heterogeneity, methodological limitations, and publication bias. The reduced variability associated with highly controlled experimental conditions in animal studies should also be considered.

Thirdly, the dose-response analysis was constrained by the dose distribution range and sample sizes of the included studies. The dose intervals reported in this study represent estimates based solely on the available evidence and should not be regarded as clinically recommended dosages.

Fourthly, the subgroup analysis results remain subject to certain limitations. Some subgroups comprised only one to three studies, resulting in insufficient statistical power and potentially unstable effect estimates. Multiple subgroup comparisons were not adjusted for multiple testing using Bonferroni correction or false discovery rate control, which may increase the risk of false positives. Furthermore, substantial heterogeneity persisted in some subgroups after stratification, indicating the potential influence of unidentified factors. Therefore, the subgroup analyses should be interpreted primarily as exploratory assessments of potential sources of heterogeneity rather than as definitive evidence.

Fifthly, significant publication bias was detected in this study. In preclinical animal research, positive results are more likely to be published than negative or null findings, which may lead to an incomplete evidence base and an inflated intervention effect (Sena et al., 2010; Ritskes-Hoitinga and Pound, 2022). Following trim-and-fill correction, the SMD for cerebral infarct volume decreased from −3.02 to −1.97, and that for NFS dropped from −1.57 to −1.06, further suggesting that the original effect sizes were likely affected by publication bias.

Sixthly, some included studies may exhibit potential data non-independence. Several studies shared highly similar experimental designs and institutional affiliations, suggesting possible overlap in research cohorts. Although sample reuse could not be verified from publicly available information, such non-independence may reduce estimates of within-group variability and increase uncertainty in pooled effect sizes. Sensitivity analyses demonstrated that sinomenine maintained a statistically significant neuroprotective effect after excluding studies with potential non-independence; however, the effect sizes for cerebral infarct volume and NFS were attenuated, indicating that the findings should be interpreted cautiously.

Finally, existing studies have primarily elucidated underlying mechanisms by identifying molecular alterations within specific signaling pathways; however, evidence supporting direct targets, upstream and downstream regulatory relationships, and crosstalk among distinct pathways remains insufficient.

### 4.6 Translational considerations and future perspectives

#### 4.6.1 Discrepancies between administration time windows and clinical treatment contexts

The translational relevance of this study is constrained by the uneven distribution of administration timing. Approximately 75% of the included studies employed pretreatment protocols, resulting in pooled effect sizes substantially influenced by pretreatment data. However, stroke onset is unpredictable, and the clinical use of neuroprotective agents is generally restricted to the acute therapeutic window after stroke onset (Paul and Candelario-Jalil, 2021; Zhang et al., 2025). Pretreatment models assess biological effects under pharmacologically protected states established prior to ischemia, which does not accurately reflect the clinical scenario of initiating treatment after stroke. Therefore, current evidence is insufficient to directly infer the clinical efficacy of sinomenine in stroke patients or to support its extrapolation to acute-phase clinical application.

#### 4.6.2 Differences between animal model characteristics and clinical patient profiles

The included studies predominantly used young, healthy male rodents, whereas clinical ischemic stroke populations are typically older and exhibit sex-related differences and comorbidities, including hypertension, diabetes, and obesity. Consequently, existing animal models are unable to adequately recapitulate the complex pathophysiological features of clinical stroke, thereby limiting the external validity of the research findings. Moreover, cerebral ischemia models differ substantially in their underlying mechanisms and the clinical stroke scenarios they represent (Matur et al., 2023; Mendes et al., 2026). All included studies employed the tMCAO model, without validation in permanent MCAO, photothrombotic, or embolic models, further restricting the generalizability of the evidence across diverse stroke subtypes.

#### 4.6.3 Inadequate pharmacological characterization and safety data

The included studies inadequately reported pharmacological information regarding sinomenine. Only one study reported drug purity, whereas the others provided no information on this parameter. Variability in drug quality may alter exposure to the active compound and its pharmacological effects, potentially contributing to inter-study heterogeneity and limiting the reproducibility and clinical translatability of the findings. Moreover, none of the included studies reported safety outcomes, and all used short-term administration regimens. Consequently, the safety of sinomenine in the context of ischemic stroke cannot be evaluated, nor can the toxicological risks, maximum tolerated dose, or therapeutic window associated with long-term administration be determined.

#### 4.6.4 Inadequate adherence to ARRIVE 2.0 and STAIR guidelines

The included studies exhibited deficiencies in experimental design, methodological reporting, and translational rigor. Based on the ARRIVE 2.0 Essential 10 items and STAIR guidelines, most studies inadequately reported key methodological parameters, including randomization procedures, blinding methods, and sample size calculations. Moreover, critical translational elements, such as therapeutic time windows, multi-model and multi-species validation, validation in aged animals, inclusion of female and comorbid animal models, and long-term functional outcome assessment, were insufficiently addressed. These shortcomings may compromise reproducibility and internal validity, thereby limiting the translational potential of sinomenine for clinical application.

Future studies should comply with the ARRIVE 2.0 and STAIR guidelines, conduct standardized, multicenter, and independently replicated validations, and report negative and neutral results to reduce publication bias. Experimental designs should incorporate post-stroke dosing paradigms that better approximate clinical practice, systematically evaluate dose-response and time-response relationships across therapeutic windows, and establish safety pharmacology parameters and long-term functional outcomes as standard observational endpoints. Validation should further encompass diverse animal models and species (e.g., gyrencephalic primates), with careful consideration of critical variables including sex, age, and comorbidities. Future research should also provide comprehensive reporting of essential drug information, including purity, source, and batch number. Mechanistic studies may integrate multi-omics approaches, including proteomics and metabolomics, to identify therapeutic targets and elucidate underlying regulatory networks.

## 5 Conclusion

This systematic review and meta-analysis provides preclinical evidence for the neuroprotective potential of sinomenine in ischemic stroke. Sinomenine alleviates cerebral ischemia-induced tissue injury and neurological dysfunction through multiple mechanisms, including suppression of neuroinflammation, enhancement of antioxidant capacity, and inhibition of neuronal apoptosis. Sinomenine dosage exhibited a significant nonlinear relationship with its neuroprotective efficacy. After adjustment for publication bias using the trim-and-fill method, the pooled effect sizes of the primary outcomes were reduced, indicating that the initial estimates may have somewhat overestimated sinomenine’s therapeutic effects. Furthermore, the included studies exhibited certain methodological limitations and inadequate reporting quality. Future research should standardize experimental designs, enhance reporting transparency, and further elucidate the underlying molecular mechanisms, thereby providing higher-quality evidence to assess the clinical translational feasibility of sinomenine as an adjunctive neuroprotective candidate for ischemic stroke.

## Data Availability

The original contributions presented in the study are included in the article/Supplementary Material, further inquiries can be directed to the corresponding authors.
