# Supplementary material for: Neuroprotective effects and mechanistic insights of sinomenine in ischemic stroke: a systematic review and meta-analysis of preclinical animal studies
Source: Front Pharmacol. 2026 Sep 22;17:1926226. doi: 10.3389/fphar.2026.1926226 (PMC13638643; doi:10.3389/fphar.2026.1926226)
Supplement: Supplementary file 1 [file Table1.docx]

**Supplementary Material**

**Supplementary Table 1:** Search strategies.

| **Database** | **Search strategy** | **Results** |
| --- | --- | --- |
| Web of science | (TS=("ischemic stroke*" OR "stroke, ischemic" OR "ischaemic stroke*" OR "stroke, ischaemic" OR "acute ischemic stroke*" OR "cryptogenic ischemic stroke*" OR "cryptogenic stroke*" OR "cryptogenic embolism stroke*" OR "wake up stroke*" OR "infarction, middle cerebral artery" OR "middle cerebral artery occlusion" OR "MCAO" OR "brain ischemia*" OR "cerebral ischemia*" OR "brain ischaemia" OR "cerebral ischaemia" OR "ischemic encephalopathy" OR "cerebral infarction*" OR "cerebral infarct*" OR "brain infarction*" OR "brain infarct*" OR "intracranial embolism" OR "cerebral embolism" OR "cerebral emboli" OR "cerebral embolus" OR "brain embolism" OR "brain emboli" OR "brain embolus" OR "cerebral ischemia reperfusion" OR "cerebral ischemic reperfusion" OR "MCAO/R" OR "cerebral ischemia reperfusion injury" OR "cerebral ischemia reperfusion damage" OR "brain ischemia reperfusion" OR "brain ischemic reperfusion" OR "brain ischemia reperfusion injury" OR "brain ischemia reperfusion damage")) AND TS=("sinomenine" OR "cucoline" OR "cuculine" OR "kukoline" OR "sinomenine a bismethyliodide" OR "sinomenine hydrochloride" OR "cucoline, hydrochloride" OR "sinomenin hydrochloride" OR "cucoline hydrochloride" OR "cocculine" OR "zhengqing fengtongning" OR "sinomenium" OR "sinomeniums" OR "sinomenium acutum" OR "sinomenium acutums" OR "acutums, sinomenium" OR "qing feng teng" OR "feng teng, qing" OR "feng tengs, qing" OR "qing feng tengs" OR "teng, qing feng" OR "tengs, qing feng" OR "sinomenii caulis") | 17 |
| PubMed | #1 ("ischemic stroke"[Mesh] OR "infarction, middle cerebral artery"[Mesh] OR "brain ischemia"[Mesh] OR "cerebral infarction"[Mesh] OR "brain infarction"[Mesh] OR "intracranial embolism"[Mesh])  #2 ("ischemic stroke*"[Title/Abstract] OR "stroke, ischemic"[Title/Abstract] OR "ischaemic stroke*"[Title/Abstract] OR "stroke, ischaemic"[Title/Abstract] OR "acute ischemic stroke*"[Title/Abstract] OR "cryptogenic ischemic stroke*"[Title/Abstract] OR "cryptogenic stroke*"[Title/Abstract] OR "cryptogenic embolism stroke*"[Title/Abstract] OR "wake up stroke*"[Title/Abstract] OR "infarction, middle cerebral artery"[Title/Abstract] OR "middle cerebral artery occlusion"[Title/Abstract] OR "MCAO"[Title/Abstract] OR "brain ischemia*"[Title/Abstract] OR "cerebral ischemia*"[Title/Abstract] OR "brain ischaemia"[Title/Abstract] OR "cerebral ischaemia"[Title/Abstract] OR "ischemic encephalopathy"[Title/Abstract] OR "cerebral infarction*"[Title/Abstract] OR "cerebral infarct*"[Title/Abstract] OR "brain infarction*"[Title/Abstract] OR "brain infarct*"[Title/Abstract] OR "intracranial embolism"[Title/Abstract] OR "cerebral embolism"[Title/Abstract] OR "cerebral emboli"[Title/Abstract] OR "cerebral embolus"[Title/Abstract] OR "brain embolism"[Title/Abstract] OR "brain emboli"[Title/Abstract] OR "brain embolus"[Title/Abstract] OR "cerebral ischemia reperfusion"[Title/Abstract] OR "cerebral ischemic reperfusion"[Title/Abstract] OR "MCAO/R"[Title/Abstract] OR "cerebral ischemia reperfusion injury"[Title/Abstract] OR "cerebral ischemia reperfusion damage"[Title/Abstract] OR "brain ischemia reperfusion"[Title/Abstract] OR "brain ischemic reperfusion"[Title/Abstract] OR "brain ischemia reperfusion injury"[Title/Abstract] OR "brain ischemia reperfusion damage"[Title/Abstract])  #3 #1 OR #2  #4 ("Sinomenium"[Mesh])  #5 ("sinomenine"[Title/Abstract] OR "cucoline"[Title/Abstract] OR "cuculine"[Title/Abstract] OR "kukoline"[Title/Abstract] OR "sinomenine a bismethyliodide"[Title/Abstract] OR "sinomenine hydrochloride"[Title/Abstract] OR "cucoline, hydrochloride"[Title/Abstract] OR "sinomenin hydrochloride"[Title/Abstract] OR "cucoline hydrochloride"[Title/Abstract] OR "cocculine"[Title/Abstract] OR "zhengqing fengtongning"[Title/Abstract] OR "sinomenium"[Title/Abstract] OR "sinomeniums"[Title/Abstract] OR "sinomenium acutum"[Title/Abstract] OR "sinomenium acutums"[Title/Abstract] OR "acutums, sinomenium"[Title/Abstract] OR "qing feng teng"[Title/Abstract] OR "feng teng, qing"[Title/Abstract] OR "feng tengs, qing"[Title/Abstract] OR "qing feng tengs"[Title/Abstract] OR "teng, qing feng"[Title/Abstract] OR "tengs, qing feng"[Title/Abstract] OR "sinomenii caulis"[Title/Abstract])  #6 #4 OR #5  #7 #3 AND #6 | 13 |
| Embase | #1 ('ischemic stroke'/exp OR 'ischemic stroke') AND [embase]/lim OR ('acute ischemic stroke'/exp OR 'acute ischemic stroke') AND [embase]/lim OR ('cryptogenic ischemic stroke'/exp OR 'cryptogenic ischemic stroke') AND [embase]/lim OR ('wake up stroke'/exp OR 'wake up stroke') AND [embase]/lim OR ('middle cerebral artery occlusion'/exp OR 'middle cerebral artery occlusion') AND [embase]/lim OR ('brain ischemia'/exp OR 'brain ischemia') AND [embase]/lim OR ('brain infarction'/exp OR 'brain infarction') AND [embase]/lim OR ('brain embolism'/exp OR 'brain embolism') AND [embase]/lim OR ('cerebral ischemia reperfusion injury'/exp OR 'cerebral ischemia reperfusion injury') AND [embase]/lim  #2 ('ischemic stroke*':ab,ti OR 'stroke, ischemic':ab,ti OR 'ischaemic stroke*':ab,ti OR 'stroke, ischaemic':ab,ti OR 'acute ischemic stroke*':ab,ti OR 'cryptogenic ischemic stroke*':ab,ti OR 'cryptogenic stroke*':ab,ti OR 'cryptogenic embolism stroke*':ab,ti OR 'cryptogenic embolic stroke':ab,ti OR 'cerebrovascular ischaemia':ab,ti OR 'cerebrovascular ischemia':ab,ti OR 'ischemic brain disease':ab,ti OR 'wake up stroke*':ab,ti OR 'infarction, middle cerebral artery':ab,ti OR 'middle cerebral artery occlusion':ab,ti OR 'mcao':ab,ti OR 'brain ischemia*':ab,ti OR 'cerebral ischemia*':ab,ti OR 'brain ischaemia':ab,ti OR 'cerebral ischaemia':ab,ti OR 'ischemic encephalopathy':ab,ti OR 'cerebral infarction*':ab,ti OR 'cerebral infarct*':ab,ti OR 'brain infarction*':ab,ti OR 'brain infarct*':ab,ti OR 'intracranial embolism':ab,ti OR 'cerebral embolism':ab,ti OR 'cerebral emboli':ab,ti OR 'cerebral embolus':ab,ti OR 'brain embolism':ab,ti OR 'brain emboli':ab,ti OR 'brain embolus':ab,ti OR 'cerebral ischemia reperfusion':ab,ti OR 'cerebral ischemic reperfusion':ab,ti OR 'mcao/r':ab,ti OR 'cerebral ischemia reperfusion injury':ab,ti OR 'cerebral ischemia reperfusion damage':ab,ti OR 'brain ischemia reperfusion':ab,ti OR 'brain ischemic reperfusion':ab,ti OR 'brain ischemia reperfusion injury':ab,ti OR 'brain ischemia reperfusion damage':ab,ti) AND [embase]/lim  #3 #1 OR #2  #4 ('sinomenine'/exp OR 'sinomenine') AND [embase]/lim OR ('sinomenine hydrochloride'/exp OR 'sinomenine hydrochloride') AND [embase]/lim OR ('zhengqing fengtongning'/exp OR 'zhengqing fengtongning') AND [embase]/lim OR ('sinomenium'/exp OR 'sinomenium') AND [embase]/lim OR ('sinomenium acutum'/exp OR 'sinomenium acutum') AND [embase]/lim OR ('sinomenii caulis'/exp OR 'sinomenii caulis') AND [embase]/lim  #5 ('sinomenine':ab,ti OR 'cucoline':ab,ti OR 'cuculine':ab,ti OR 'kukoline':ab,ti OR 'sinomenine a bismethyliodide':ab,ti OR 'sinomenine hydrochloride':ab,ti OR 'cucoline, hydrochloride':ab,ti OR 'sinomenin hydrochloride':ab,ti OR 'cucoline hydrochloride':ab,ti OR 'cocculine':ab,ti OR 'zhengqing fengtongning':ab,ti OR 'sinomenium':ab,ti OR 'sinomeniums':ab,ti OR 'sinomenium acutum':ab,ti OR 'sinomenium acutums':ab,ti OR 'acutums, sinomenium':ab,ti OR 'qing feng teng':ab,ti OR 'feng teng, qing':ab,ti OR 'feng tengs, qing':ab,ti OR 'qing feng tengs':ab,ti OR 'teng, qing feng':ab,ti OR 'tengs, qing feng':ab,ti OR 'sinomenii caulis':ab,ti) AND [embase]/lim  #6 #4 OR #5  #7 #3 AND #6 | 41 |
| Cochrane Library | #1 MeSH descriptor: [Ischemic Stroke] explode all trees  #2 MeSH descriptor: [Infarction, Middle Cerebral Artery] explode all trees  #3 MeSH descriptor: [Brain Ischemia] explode all trees  #4 MeSH descriptor: [Cerebral Infarction] explode all trees  #5 MeSH descriptor: [Brain Infarction] explode all trees  #6 MeSH descriptor: [Intracranial Embolism] explode all trees  #7 #1 OR #2 OR #3 OR #4 OR #5 OR #6  #8 ((ischemic NEXT stroke*) OR "stroke, ischemic" OR (ischaemic NEXT stroke*) OR "stroke, ischaemic" OR (acute NEXT ischemic NEXT stroke*) OR (cryptogenic NEXT ischemic NEXT stroke*) OR (cryptogenic NEXT stroke*) OR (cryptogenic NEXT embolism NEXT stroke*) OR (wake NEXT up NEXT stroke*) OR "infarction, middle cerebral artery" OR "middle cerebral artery occlusion" OR "MCAO" OR (brain NEXT ischemia*) OR (cerebral NEXT ischemia*) OR "brain ischaemia" OR "cerebral ischaemia" OR "ischemic encephalopathy" OR (cerebral NEXT infarction*) OR (cerebral NEXT infarct*) OR (brain NEXT infarction*) OR (brain NEXT infarct*) OR "intracranial embolism" OR "cerebral embolism" OR "cerebral emboli" OR "cerebral embolus" OR "brain embolism" OR "brain emboli" OR "brain embolus" OR "cerebral ischemia reperfusion" OR "cerebral ischemic reperfusion" OR "MCAO/R" OR "cerebral ischemia reperfusion injury" OR "cerebral ischemia reperfusion damage" OR "brain ischemia reperfusion" OR "brain ischemic reperfusion" OR "brain ischemia reperfusion injury" OR "brain ischemia reperfusion damage"):ti,ab,kw  (Word variations have been searched)  #9 #7 OR #8  #10 MeSH descriptor: [Sinomenium] explode all trees  #11 ("sinomenine" OR "cucoline" OR "cuculine" OR "kukoline" OR "sinomenine a bismethyliodide" OR "sinomenine hydrochloride" OR "cucoline, hydrochloride" OR "sinomenin hydrochloride" OR "cucoline hydrochloride" OR "cocculine" OR "zhengqing fengtongning" OR "sinomenium" OR "sinomeniums" OR "sinomenium acutum" OR "sinomenium acutums" OR "acutums, sinomenium" OR "qing feng teng" OR "feng teng, qing" OR "feng tengs, qing" OR "qing feng tengs" OR "teng, qing feng" OR "tengs, qing feng" OR "sinomenii caulis"):ti,ab,kw  (Word variations have been searched)  #12 #10 OR #11  #13 #9 AND #12 | 0 |
| CNKI | SU%=('卒中' + '中风' + '脑梗死' + '脑缺血' + '脑梗塞' + '脑栓塞' + '脑血管意外' + '缺血性脑血管病' + '脑血栓' + '脑缺血再灌注' + '脑缺血/再灌注' + 'MCAO' + 'MCAO/R' + 'CIRI') * ('青藤碱' + '正清风痛宁' + '正清风痛宁缓释片' + '盐酸青藤碱' + '青风藤') | 68 |
| Wanfang | 主题:(("卒中" OR "中风" OR "脑梗死" OR "脑缺血" OR "脑梗塞" OR "脑栓塞" OR "脑血管意外" OR "缺血性脑血管病" OR "脑血栓" OR "脑缺血再灌注" OR "脑缺血/再灌注" OR "MCAO" OR "MCAO/R" OR "CIRI") AND ("青藤碱" OR "正清风痛宁" OR "正清风痛宁缓释片" OR "盐酸青藤碱" OR "青风藤")) | 41 |
| VIP | M=(("卒中" OR "中风" OR "脑梗死" OR "脑缺血" OR "脑梗塞" OR "脑栓塞" OR "脑血管意外" OR "缺血性脑血管病" OR "脑血栓" OR "脑缺血再灌注" OR "脑缺血/再灌注" OR "MCAO" OR "MCAO/R" OR "CIRI") AND ("青藤碱" OR "正清风痛宁" OR "正清风痛宁缓释片" OR "盐酸青藤碱" OR "青风藤")) | 26 |
| Sinomed | (("卒中"[不加权:扩展]) OR ("卒中"[常用字段:智能] OR "中风"[常用字段:智能] OR "脑梗死"[常用字段:智能] OR "脑缺血"[常用字段:智能] OR "脑梗塞"[常用字段:智能] OR "脑栓塞"[常用字段:智能] OR "脑血管意外"[常用字段:智能] OR "缺血性脑血管病"[常用字段:智能] OR "脑血栓"[常用字段:智能] OR "脑缺血再灌注"[常用字段:智能] OR "脑缺血/再灌注"[常用字段:智能] OR "MCAO"[常用字段:智能] OR "MCAO/R"[常用字段:智能] OR "CIRI"[常用字段:智能])) AND (("青藤碱"[不加权:扩展]) OR ("青藤碱"[常用字段:智能] OR "正清风痛宁"[常用字段:智能] OR "正清风痛宁缓释片"[常用字段:智能] OR "盐酸青藤碱"[常用字段:智能] OR "青风藤"[常用字段:智能])) | 52 |

**Supplementary Table 2:** Sinomenine source and purity information.

| **Study** | **Source/supplier** | **Purity** | **Solvent** |
| --- | --- | --- | --- |
| Feng (2010) | Hunan Zhengqing Pharmaceutical Co., Ltd. | NR | NR |
| Li (2010) | Hunan Zhengqing Pharmaceutical Co., Ltd. | NR | NR |
| Li (2009) | Hunan Zhengqing Pharmaceutical Co., Ltd. | NR | NR |
| Liang (1999) | Xi’an Pharmaceutical Factory | NR | Saline |
| Wu (2010a) | Hunan Zhengqing Pharmaceutical Co., Ltd. | NR | NR |
| Zhou (2009a) | Hunan Zhengqing Pharmaceutical Co., Ltd. | NR | NR |
| Zhou (2009b) | Hunan Zhengqing Pharmaceutical Co., Ltd. | NR | NR |
| Bi (2021) | MedChemExpress | NR | NR |
| Li (2026) | NR | NR | 10% dimethyl sulfoxide, 40% polyethylene glycol 300, 5% Tween 80 and 45% saline |
| Qiu (2016a) | Purchased from Selleck | NR | NR |
| Qiu (2016b) | NR | NR | NR |
| Wu (2011) | National Institute for the Control of Pharmaceutical and Biological Products (Beijing, China) | Above 98% | Distilled water |
| Yang (2016) | NR | NR | NR |
| Li (2008) | Hunan Zhengqing Pharmaceutical Co., Ltd. | NR | NR |
| Wu (2010b) | Hunan Zhengqing Pharmaceutical Co., Ltd. | NR | NR |

NR, not reported.

**Supplementary Table 3:** Assessment of reporting quality of included studies based on the ARRIVE 2.0 Essential 10 guidelines.

| **Study** | **A** | **B** | **C** | **D** | **E** | **F** | **G** | **H** | **I** | **J** | **K** | **L** | **M** | **N** | **O** | **P** | **Q** | **R** | **S** | **T** | **U** | **V** |
| --- | --- | --- | --- | --- | --- | --- | --- | --- | --- | --- | --- | --- | --- | --- | --- | --- | --- | --- | --- | --- | --- | --- |
| Feng (2010) | Y | Y | Y | NR | Y | Y | Y | Y | NR | NR | Y | NR | Y | NR | Y | Y | Y | Y | NR | Y | Y | NR |
| Li (2010) | Y | Y | Y | NR | Y | Y | Y | Y | NR | NR | Y | NR | Y | NR | Y | Y | Y | Y | NR | Y | Y | NR |
| Li (2009) | Y | Y | Y | NR | Y | Y | Y | Y | NR | NR | Y | NR | Y | NR | Y | Y | Y | Y | NR | Y | Y | NR |
| Liang (1999) | Y | Y | Y | NR | Y | NR | Y | Y | NR | NR | Y | NR | Y | NR | Y | Y | Y | Y | NR | Y | Y | NR |
| Wu (2010a) | Y | Y | Y | NR | Y | NR | Y | Y | NR | NR | Y | NR | Y | NR | Y | Y | Y | Y | NR | Y | Y | NR |
| Zhou (2009a) | Y | Y | Y | NR | Y | Y | Y | Y | NR | NR | Y | NR | Y | NR | Y | Y | Y | Y | NR | Y | Y | NR |
| Zhou (2009b) | Y | Y | Y | NR | Y | NR | Y | Y | NR | NR | Y | NR | Y | NR | Y | Y | Y | Y | NR | Y | Y | NR |
| Bi (2021) | Y | Y | Y | NR | NR | NR | Y | NR | NR | NR | Y | NR | Y | NR | Y | Y | Y | Y | NR | Y | Y | NR |
| Li (2026) | Y | Y | Y | Y | Y | Y | Y | Y | NR | Y | Y | NR | Y | Y | Y | Y | Y | Y | Y | Y | Y | NR |
| Qiu (2016a) | Y | Y | Y | NR | Y | NR | Y | Y | NR | Y | Y | NR | Y | NR | Y | Y | Y | Y | NR | Y | Y | NR |
| Qiu (2016b) | Y | Y | Y | NR | Y | NR | Y | NR | NR | Y | Y | NR | Y | NR | Y | Y | Y | Y | NR | Y | Y | NR |
| Wu (2011) | Y | Y | Y | NR | Y | Y | Y | Y | NR | Y | Y | NR | Y | NR | Y | Y | Y | Y | NR | Y | Y | NR |
| Yang (2016) | Y | Y | Y | NR | Y | Y | Y | Y | NR | NR | Y | NR | Y | NR | Y | NR | Y | Y | Y | Y | Y | NR |
| Li (2008) | Y | Y | Y | NR | Y | Y | Y | Y | NR | NR | Y | NR | Y | NR | Y | Y | Y | Y | NR | Y | Y | NR |
| Wu (2010b) | Y | Y | Y | NR | Y | NR | Y | Y | NR | NR | Y | NR | Y | NR | Y | Y | Y | Y | NR | Y | Y | NR |

Y, yes (item adequately reported); NR, not reported. A, Study design (a): The groups being compared, including control groups. If no control group has been used, the rationale should be stated; B, Study design (b): The experimental unit (e.g., a single animal, litter, or cage of animals); C, Sample size (a): Specify the exact number of experimental units allocated to each group, and the total number in each experiment. Also indicate the total number of animals used; D, Sample size (b): Explain how the sample size was decided. Provide details of any a priori sample size calculation, if done; E, Inclusion and exclusion criteria (a): Describe any criteria used for including and excluding animals (or experimental units) during the experiment, and data points during the analysis. Specify if these criteria were established a priori. If no criteria were set, state this explicitly; F, Inclusion and exclusion criteria (b): For each experimental group, report any animals, experimental units, or data points not included in the analysis and explain why. If there were no exclusions, state so; G, Inclusion and exclusion criteria (c): For each analysis, report the exact value of n in each experimental group; H, Randomisation (a): State whether randomisation was used to allocate experimental units to control and treatment groups. If done, provide the method used to generate the randomisation sequence; I, Randomisation (b): Describe the strategy used to minimise potential confounders such as the order of treatments and measurements, or animal/cage location. If confounders were not controlled, state this explicitly; J, Blinding: (Describe who was aware of the group allocation at the different stages of the experiment (during the allocation, the conduct of the experiment, the outcome assessment, and the data analysis); K, Outcome measures (a): Clearly define all outcome measures assessed (e.g., cell death, molecular markers, or behavioural changes); L, Outcome measures (b): For hypothesis-testing studies, specify the primary outcome measure, i.e., the outcome measure that was used to determine the sample size; M, Statistical methods (a): Provide details of the statistical methods used for each analysis, including software used; N, Statistical methods (b): Describe any methods used to assess whether the data met the assumptions of the statistical approach, and what was done if the assumptions were not met; O, Experimental animals (a): Provide species-appropriate details of the animals used, including species, strain and substrain, sex, age or developmental stage, and, if relevant, weight; P, Experimental animals (b): Provide further relevant information on the provenance of animals, health/immune status, genetic modification status, genotype, and any previous procedures; Q, Experimental procedures (a): What was done, how it was done, and what was used; R, Experimental procedures (b): When and how often; S, Experimental procedures (c): Where (including detail of any acclimatisation periods); T, Experimental procedures (d): Why (provide rationale for procedures); U, Results (a): Summary/descriptive statistics for each experimental group, with a measure of variability where applicable (e.g., mean and SD, or median and range); V, Results (b): If applicable, the effect size with a confidence interval.

**Supplementary Table 4:** Assessment of translational readiness of included studies based on the STAIR criteria.

| **Study** | **A** | **B** | **C** | **D** | **E** | **F** | **G** | **H** | **I** | **J** | **K** | **L** | **M** | **N** | **O** | **P** | **Q** |
| --- | --- | --- | --- | --- | --- | --- | --- | --- | --- | --- | --- | --- | --- | --- | --- | --- | --- |
| Feng (2010) | Y | N | N | Y | NR | NR | Y | Y | NR | N | N | N | N | N | N | Y | NR |
| Li (2010) | Y | N | N | Y | NR | Y | Y | Y | NR | N | N | N | N | N | N | Y | NR |
| Li (2009) | Y | N | N | Y | NR | NR | Y | Y | NR | N | N | N | N | N | N | Y | NR |
| Liang (1999) | Y | N | N | Y | NR | Y | Y | NR | NR | N | N | N | N | N | N | N | NR |
| Wu (2010a) | Y | N | N | Y | NR | NR | Y | NR | NR | N | N | N | N | Y | N | Y | NR |
| Zhou (2009a) | Y | N | N | Y | NR | NR | Y | Y | NR | N | N | N | N | Y | N | Y | NR |
| Zhou (2009b) | Y | N | N | Y | NR | NR | Y | NR | NR | N | N | N | N | Y | N | Y | NR |
| Bi (2021) | N | N | N | NR | NR | NR | NR | NR | NR | N | N | N | N | N | N | Y | Y |
| Li (2026) | N | N | N | Y | Y | NR | Y | Y | Y | N | N | N | N | Y | N | Y | Y |
| Qiu (2016a) | Y | N | N | Y | Y | Y | Y | NR | NR | N | N | N | N | N | N | Y | Y |
| Qiu (2016b) | Y | N | N | NR | Y | NR | Y | NR | NR | N | N | NR | N | N | N | Y | Y |
| Wu (2011) | Y | Y | N | Y | Y | Y | Y | Y | NR | N | N | N | N | N | N | Y | Y |
| Yang (2016) | N | N | N | Y | NR | Y | Y | Y | NR | N | N | N | N | N | N | Y | NR |
| Li (2008) | Y | N | N | Y | NR | NR | Y | Y | NR | N | N | N | N | N | N | Y | NR |
| Wu (2010b) | Y | N | N | Y | NR | NR | Y | NR | NR | N | N | N | N | Y | N | Y | NR |

Y, yes (criterion fulfilled); N, no (criterion not fulfilled); NR, not reported. A, Adequate dose-response curve; B, Define therapeutic time window; C, Transient and permanent ischemia models; D, Report randomization; E, Implement blinding; F, Perform physiological monitoring; G, Preset inclusion/exclusion criteria; H, Report excluded animals; I, Perform sample size and power calculation; J, Assess acute and long-term histological and functional outcomes; K, Validate in multiple species; L, Validate in both sexes; M, Validate in aged animals; N, Validate in comorbid models; O, Validate reproducibility; P, Use clinically relevant biomarkers; Q, Disclose conflicts of interest.

**Supplementary Table 5:** Subgroup analysis of cerebral infarct volume.

| **Subgroup** | **Study** | **SMD (95% CI)** | **Heterogeneity *I^2^* (%)** | ***P* value** | **Subgroup (*P* value)** |
| --- | --- | --- | --- | --- | --- |
| Animal strain |  |  |  |  | < 0.00001 |
| C57BL/6 mice | 4 | -1.20 (-1.92, -0.48) | 0 | 0.51 |  |
| SPD rats | 7 | -2.69 (-4.05, -1.34) | 81 | < 0.0001 |  |
| Wistar rats | 6 | -5.61 (-7.02, -4.19) | 0 | 0.70 |  |
| Comorbidity status |  |  |  |  | 0.001 |
| Healthy animals | 13 | -2.37 (-3.27, -1.47) | 74 | < 0.00001 |  |
| Diabetic animals | 4 | -5.65 (-7.41, -3.90) | 0 | 0.50 |  |
| Experimental blinding |  |  |  |  | 0.44 |
| Blinded studies | 7 | -2.65 (-4.07, -1.23) | 74 | 0.0008 |  |
| Non-blinded studies | 10 | -3.42 (-4.77, -2.07) | 80 | < 0.00001 |  |
| Dosage |  |  |  |  | 0.05 |
| 10 mg/kg | 4 | -1.27 (-2.66, 0.12) | 74 | 0.008 |  |
| 20 mg/kg | 2 | -1.85 (-2.99, -0.70) | 0 | 0.95 |  |
| 30 mg/kg | 6 | -4.31 (-6.43, -2.18) | 77 | 0.0006 |  |
| 60 mg/kg | 4 | -5.70 (-9.38, -2.03) | 79 | 0.002 |  |
| 90 mg/kg | 1 | -2.57 (-4.26, -0.88) | - | - |  |
| Timing of administration |  |  |  |  | 0.005 |
| Pre-MCAO | 12 | -3.94 (-5.30, -2.58) | 81 | < 0.00001 |  |
| Post-MCAO | 5 | -1.56 (-2.53, -0.59) | 44 | 0.13 |  |

SPD rats, Sprague-Dawley rats; MCAO, middle cerebral artery occlusion.

**Supplementary Table 6:** Subgroup analysis of NFS.

| **Subgroup** | **Study** | **SMD (95% CI)** | **Heterogeneity *I^2^* (%)** | ***P* value** | **Subgroup (*P* value)** |
| --- | --- | --- | --- | --- | --- |
| Animal strain |  |  |  |  | 0.07 |
| C57BL/6 mice | 4 | -0.93 (-1.46, -0.40) | 0 | 0.52 |  |
| SPD rats | 6 | -1.96 (-3.08, -0.83) | 80 | 0.0002 |  |
| Wistar rats | 2 | -2.21 (-3.46, -0.96) | 0 | 0.67 |  |
| Experimental blinding |  |  |  |  | 0.05 |
| Blinded studies | 6 | -0.98 (-1.46, -0.51) | 0 | 0.57 |  |
| Non-blinded studies | 6 | -2.19 (-3.33, -1.05) | 79 | 0.0003 |  |
| Dosage |  |  |  |  | 0.0001 |
| 10 mg/kg | 3 | -0.47 (-1.02, 0.09) | 0 | 0.71 |  |
| 20 mg/kg | 2 | -1.29 (-2.08, -0.51) | 0 | 0.47 |  |
| 30 mg/kg | 3 | -3.11 (-4.32, -1.89) | 20 | 0.29 |  |
| 60 mg/kg | 2 | -2.58 (-3.63, -1.53) | 0 | 0.36 |  |
| 80 mg/kg | 1 | -0.85 (-2.06, 0.35) | - | - |  |
| 90 mg/kg | 1 | -1.89 (-2.78, -1.01) | - | - |  |
| Timing of administration |  |  |  |  | 0.09 |
| Pre-MCAO | 7 | -1.98 (-2.96, -0.99) | 76 | 0.0003 |  |
| Post-MCAO | 5 | -1.01 (-1.52, -0.49) | 0 | 0.43 |  |

SPD rats, Sprague-Dawley rats; MCAO, middle cerebral artery occlusion.

**Supplementary Table 7:** Subgroup analysis of brain water content.

| **Subgroup** | **Study** | **SMD (95% CI)** | **Heterogeneity *I^2^* (%)** | ***P* value** | **Subgroup (*P* value)** |
| --- | --- | --- | --- | --- | --- |
| Animal strain |  |  |  |  | 0.10 |
| C57BL/6 mice | 5 | -1.54 (-2.34, -0.75) | 24 | 0.26 |  |
| SPD rats | 4 | -3.09 (-5.08, -1.09) | 86 | < 0.0001 |  |
| Wistar rats | 4 | -3.14 (-4.63, -1.65) | 42 | 0.16 |  |
| Experimental blinding |  |  |  |  | 0.08 |
| Blinded studies | 4 | -1.54 (-2.59, -0.49) | 41 | 0.17 |  |
| Non-blinded studies | 9 | -2.91 (-3.99, -1.83) | 72 | 0.0004 |  |
| Dosage |  |  |  |  | 0.002 |
| 10 mg/kg | 3 | -0.97 (-1.65, -0.30) | 0 | 0.90 |  |
| 20 mg/kg | 3 | -2.11 (-3.27, -0.96) | 25 | 0.26 |  |
| 30 mg/kg | 3 | -2.83 (-4.08, -1.57) | 35 | 0.22 |  |
| 60 mg/kg | 3 | -5.03 (-7.33, -2.73) | 56 | 0.10 |  |
| 90 mg/kg | 1 | -2.20 (-3.77, -0.64) | - | - |  |
| Timing of administration |  |  |  |  | 0.04 |
| Pre-MCAO | 8 | -3.13 (-4.38, -1.88) | 75 | 0.0002 |  |
| Post-MCAO | 5 | -1.54 (-2.34, -0.75) | 24 | 0.26 |  |

SPD rats, Sprague-Dawley rats; MCAO, middle cerebral artery occlusion.

**Supplementary Table 8:** Results of publication bias assessment.

| **Outcomes** | **Funnel plot** | **Egger’s test (*P* value)** | **Trim-and-fill imputed studies (n)** | **Before trim-and-fill analysis (95% CI)** | **After trim-and-fill analysis (95% CI)** |
| --- | --- | --- | --- | --- | --- |
| Cerebral infarct volume | Asymmetric | < 0.001 | 6 | -3.02 (-3.96, -2.08) | -1.97 (-2.93, -1.01) |
| NFS | Asymmetric | 0.023 | 4 | -1.57 (-2.18, -0.97) | -1.06 (-1.72, -0.40) |
| Brain water content | Asymmetric | < 0.001 | 3 | -2.47 (-3.28, -1.66) | -1.93 (-2.83, -1.04) |

**Supplementary Figure 1.** Subgroup analysis of cerebral infarct volume by animal strain.

**Supplementary Figure 2.** Subgroup analysis of cerebral infarct volume by comorbidity status.

**Supplementary Figure 3.** Subgroup analysis of cerebral infarct volume by dosage.

**Supplementary Figure 4.** Subgroup analysis of cerebral infarct volume by timing of administration.

**Supplementary Figure 5.** Subgroup analysis of cerebral infarct volume by experimental blinding.

**Supplementary Figure 6.** Subgroup analysis of NFS by animal strain.

**Supplementary Figure 7.** Subgroup analysis of NFS by experimental blinding.

**Supplementary Figure 8.** Subgroup analysis of NFS by dosage.

**Supplementary Figure 9.** Subgroup analysis of NFS by timing of administration.

**Supplementary Figure 10.** Subgroup analysis of brain water content by animal strain.

**Supplementary Figure 11.** Subgroup analysis of brain water content by experimental blinding.

**Supplementary Figure 12.** Subgroup analysis of brain water content by dosage.

**Supplementary Figure 13.** Subgroup analysis of brain water content by timing of administration.

**Supplementary Figure 14.** Leave-one-out sensitivity analysis for outcomes. (A) Sensitivity analysis of cerebral infarct volume. (B) Sensitivity analysis of NFS. (C) Sensitivity analysis of brain water content.

**Supplementary Figure 15.** Sensitivity analysis forest plot for cerebral infarct volume after excluding potentially non-independent studies.

**Supplementary Figure 16.** Sensitivity analysis forest plot for NFS after excluding potentially non-independent studies.

**Supplementary Figure 17.** Trim-and-fill analysis for outcomes. (A) Funnel plot of cerebral infarct volume after trim-and-fill. (B) Funnel plot of NFS after trim-and-fill. (C) Funnel plot of brain water content after trim-and-fill.
